# The RanBP2/RanGAP1*SUMO1/Ubc9 SUMO E3 ligase is a disassembly machine for Crm1-dependent nuclear export complexes

**DOI:** 10.1038/ncomms11482

**Published:** 2016-05-10

**Authors:** Tobias Ritterhoff, Hrishikesh Das, Götz Hofhaus, Rasmus R. Schröder, Annette Flotho, Frauke Melchior

**Affiliations:** 1 Zentrum für Molekulare Biologie der Universität Heidelberg, DKFZ-ZMBH Alliance, Heidelberg 69120, Germany; 2 Department of Biochemistry, University of Washington, Seattle, Washington 98195, USA; 3 Cryo Electron Microscopy, CellNetworks, BioQuant, Universitätsklinikum Heidelberg, Heidelberg 69120, Germany

## Abstract

Continuous cycles of nucleocytoplasmic transport require disassembly of transport receptor/Ran-GTP complexes in the cytoplasm. A basic disassembly mechanism in all eukaryotes depends on soluble RanGAP and RanBP1. In vertebrates, a significant fraction of RanGAP1 stably interacts with the nucleoporin RanBP2 at a binding site that is flanked by FG-repeats and Ran-binding domains, and overlaps with RanBP2's SUMO E3 ligase region. Here, we show that the RanBP2/RanGAP1*SUMO1/Ubc9 complex functions as an autonomous disassembly machine with a preference for the export receptor Crm1. We describe three *in vitro* reconstituted disassembly intermediates, which show binding of a Crm1 export complex via two FG-repeat patches, cargo-release by RanBP2's Ran-binding domains and retention of free Crm1 at RanBP2 after Ran-GTP hydrolysis. Intriguingly, all intermediates are compatible with SUMO E3 ligase activity, suggesting that the RanBP2/RanGAP1*SUMO1/Ubc9 complex may link Crm1- and SUMO-dependent functions.

## Abstract (web summary)

Continuous cycles of nucleo-cytoplasmic transport require disassembly of transport receptor-Ran-GTP complexes in the cytoplasm. Here the authors elucidate the specific function of the RanBP2 complex in the disassembly process.

## Graphical abstract

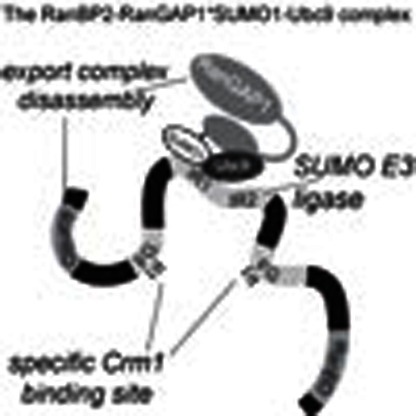

Directionality of nucleocytoplasmic transport is controlled by spatially separated assembly and disassembly of transport receptor/cargo complexes^1^,^2^,^3^. Key players in these pathways are nuclear import and export receptors of the importin β/karyopherin β-superfamily, the GTPase Ran and regulatory components of the Ran GTPase cycle. These include a nuclear exchange factor, a cytoplasmic GTPase-activating protein (RanGAP1 in vertebrates) and proteins with high-affinity Ran-GTP-binding domains (RanBDs) such as RanBP1. While import receptors associate with Ran-GTP to dissociate their cargo in the nucleus, export receptors require Ran-GTP to form cargo-containing export complexes. Common to both receptor types is that they leave the nucleus in association with Ran-GTP (trimeric export complexes and recycling import receptor/Ran-GTP complexes), and that they dissociate from their interacting partners in the cytoplasm on RanGAP-induced Ran-GTP hydrolysis. While import receptors then engage with novel cargo, export receptors recycle back to the nucleus. Spatially separated assembly and disassembly of transport receptor/cargo complexes also contributes to mitotic pathways such as the assembly of the mitotic spindle^4^,^5^,^6^,^7^,^8^.

A basic disassembly-machinery, which is made up of soluble RanGAP and RanBP1, is conserved among all eukaryotes. It is required for the disassembly of both trimeric export complexes and recycling import/Ran-GTP complexes. Transport receptors protect Ran-GTP from the action of RanGAP, unless simultaneous binding of the cytoplasmic RanBP1 to Ran-GTP lifts this protection^1^,^2^,^3^,^9^,^10^,^11^. In addition, the RanBD/Ran-GTP interaction was shown to promote cargo-release from export complexes containing the prototypic export receptor Crm1 (also known as exportin1), which precedes GTP-hydrolysis^4^,^6^,^8^,^12^,^13^,^14^,^15^.

In vertebrates, a significant fraction of RanGAP1 resides at nuclear pore complexes^9^,^11^,^16^,^17^, where it forms a stable complex with the 358 kDa vertebrate-specific and essential nucleoporin RanBP2 (also known as Nup358; Fig. 1)^12^,^14^,^15^,^18^,^19^. RanBP2 has been implicated in numerous transport pathways involving, for example, importin α/β^16^,^17^,^20^,^21^, transportin 1 (refs 18, 21, 22, 23), transportin 3 (refs 20, 23, 24), CAS^22^,^25^, importin 7 (ref. 24) and Crm1 (refs 26, 27, 28), contributes to mitotic processes^29^ and is a SUMO E3 ligase^17^,^19^,^30^.

RanBP2's interaction with RanGAP1 depends on post-translational modification of the C-terminal tail domain of RanGAP1 with the ubiquitin-related modifier SUMO1 (refs 9, 11), and requires the SUMO E2 conjugating enzyme Ubc9 (ref. 31). The latter forms a molecular glue between SUMOylated RanGAP1 and the internal repeat (IR) region of RanBP2 (RanBP2/RanGAP1*SUMO1/Ubc9 complex=RanBP2 complex, see Fig. 1)^17^,^32^. In rapidly dividing tissue culture cells, which express an excess of RanGAP1 compared with RanBP2, all RanBP2 seems engaged in this stable complex^2^,^5^,^17^.

RanBP2 contains numerous FG-repeats, which serve as low-affinity binding sites for nuclear transport receptors of the importin β-superfamily^1^,^2^,^3^,^5^,^6^,^7^, and four RanBDs, which in isolation are equivalent to that of RanBP1 (refs 1, 2, 3, 4, 6, 7, 8, 10). With its RanBDs and the associated RanGAP1*SUMO1, the RanBP2 complex contains the two elements that are required for disassembly of transport receptor/Ran-GTP complexes, and may thus represent an alternative to soluble RanGAP1 and RanBP1 with redundant, partially overlapping and/or with highly specific functions. First biochemical insights for specific interactions of RanBP2 with recycling importin β/Ran-GTP complexes were provided using purified rat liver RanBP2 (refs 4, 6, 8, 9, 10, 11, 13) and GST-RanBP2 fragments^9^,^11^,^12^,^13^,^14^,^15^,^17^, but due to the unavailability of a recombinant RanBP2 complex, its role in transport complex disassembly has never been molecularly tested.

We recently succeeded in reconstituting a 180 kDa RanBP2 complex *in vitro* (Fig. 1)^12^,^14^,^15^,^16^,^17^,^19^. This part of the native 450 kDa RanBP2 complex incorporates two RanBDs, full-length RanGAP1*SUMO1, two FG-repeat patches and is active as a SUMO E3 ligase. It allowed us to address the question, whether the RanBP2 complex binds specific transport receptors and transport receptor complexes in proximity to RanGAP1. Indeed, the recombinant RanBP2 complex shows striking binding preference for the export receptor Crm1 compared with several other transport receptors. Stable interaction with Crm1 and with Crm1-containing export complexes requires both FG-repeat patches of the recombinant RanBP2 complex. This leads to a structural rearrangement of the largely unfolded RanBP2 fragment^16^,^17^,^18^,^19^,^21^, and results in a molecular embrace with Crm1. Importantly, the RanBP2 complex can disassemble bound Crm1-dependent export complexes *in vitro*, revealing that neither soluble RanGAP1 nor RanBP1 are *per se* required for this process. Intriguingly, the SUMO E3 ligase activity of the RanBP2 complex is retained on Crm1-interaction. These findings allow speculating that Crm1-dependent processes in interphase and/or in mitosis are directly linked to RanBP2-dependent SUMOylation.

## Results

The aim of this study was to gain insights into molecular functions of the C-terminal part of the human RanBP2 complex that includes RanGAP activity, RanBDs and FG-repeats. For this, we made use of an *in vitro* reconstituted complex that consists of a C-terminally His-tagged 86 kDa RanBP2 fragment spanning RanBD3 and 4 (RanBP2-3/4 fragment), untagged, full-length Ubc9 and *in vitro* SUMOylated RanGAP1 (refs 17, 18, 20, 23).

### The RanBP2-3/4 complex interacts stably with Crm1

To test, whether the RanBP2-3/4 complex can directly interact with transport receptors, we purified the import receptors importin β^20^,^21^,^22^, transportin 1 (refs 22, 23, 24) and importin 13 (refs 24, 25), the export receptors Crm1 (refs 33, 34, 35) and CAS (the export receptor of importin-α^36^), and the structurally unrelated import receptor for Ran-GDP, NTF2 (refs 37, 38). We incubated these receptors at a 2 μM concentration with 1 μM RanBP2-3/4 complex and applied the samples to gel filtration on a Superdex200 column (3 ml bed volume, 15 min run duration). In gel filtration chromatography, the RanBP2-3/4 complex displayed a large Stokes radius (Supplementary Fig. 1a), which allowed us to distinguish it from recombinant transport receptors in the elution profiles (possible oligomerization of the complex was excluded by size exclusion chromatography with multi-angle light scattering analysis—SEC-MALS, Supplementary Fig. 1d). To test for interactions, column peak fractions were analysed by SDS-PAGE (Fig. 2a; right panels show peak fractions). In this setting, Crm1 was the only transport receptor that significantly co-eluted with the RanBP2-3/4 complex (Fig. 2a,b). Stable interaction of Crm1 was also observed for the free RanBP2-3/4 fragment (Supplementary Fig. 1b). These findings advocate an unexpectedly high-affinity interaction^39^,^40^ between the export receptor Crm1 and the nucleoporin RanBP2.

### Both FG-patches in RanBP2-3/4 are required for interaction

Given the two FG-repeat patches flanking the IR-region of RanBP2, we wondered whether they act as binding sites for Crm1. To test this, we created RanBP2-3/4 complex variants in which the FG-repeat patches were non-functional due to phenylalanine-to-serine mutations (Fig. 3a)^41^. Indeed, destruction of these patches either individually or in combination caused a complete loss of Crm1 co-migration (Fig. 3b, RanBP2-3/4 mFG1, mFG2 and mFG1/2 complexes). This suggested that both FG-repeat patches are required for stable Crm1-binding.

Two recent crystal structures provide detailed insights into Crm1/FG repeat interactions: a structure of the yeast Crm1 homologue Xpo1p in complex with Ran (Gsp1p-GTP) and the FG-containing Yrb2p (homologue of human RanBP3)^42^, and a structure of the human FG-nucleoporin Nup214 bound to a Crm1 export complex^43^. Altogether, these studies revealed three conserved hydrophobic FG-binding pockets at the termini of Crm1. To address whether these pockets are also involved in binding of RanBP2, we generated numerous Crm1 variants. Unfortunately, most of these were insoluble, similar to what has been observed by others^43^. However, we were able to purify a Crm1 variant with one point mutation each in two of the conserved FG-binding pockets (Crm1-Y105A/W880A). This Crm1 double-mutant showed a slight, yet reproducible binding defect with the RanBP2-3/4 complex in gel filtration assays (Fig. 3c), indicating that these pockets also contribute to RanBP2-3/4 interaction. Whether additional binding pockets for RanBP2 exist on Crm1 will have to await further investigations including structural analyses.

**Figure 1:**
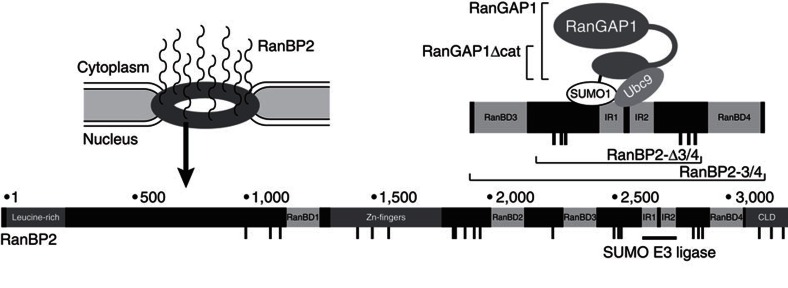
RanBP2 and the RanBP2 complex. Schematic view of a nuclear pore complex (upper left), of the domain structure of RanBP2 (bottom) and of the RanBP2/RanGAP1*SUMO1/Ubc9 complex (RanBP2 complex, upper right). RanGAP1 is depicted with its N-terminal catalytic domain (large oval), its C-terminal tail domain (Δcat, small oval) and the acidic linker region (arch). Reconstituted versions of the RanBP2 complex used in this work are indicated. CLD, cyclophilin-like domain; IR, internal repeat region of RanBP2 containing its SUMO E3 ligase actvity. Vertical dashes denotes FG-repeats.

**Figure 2:**
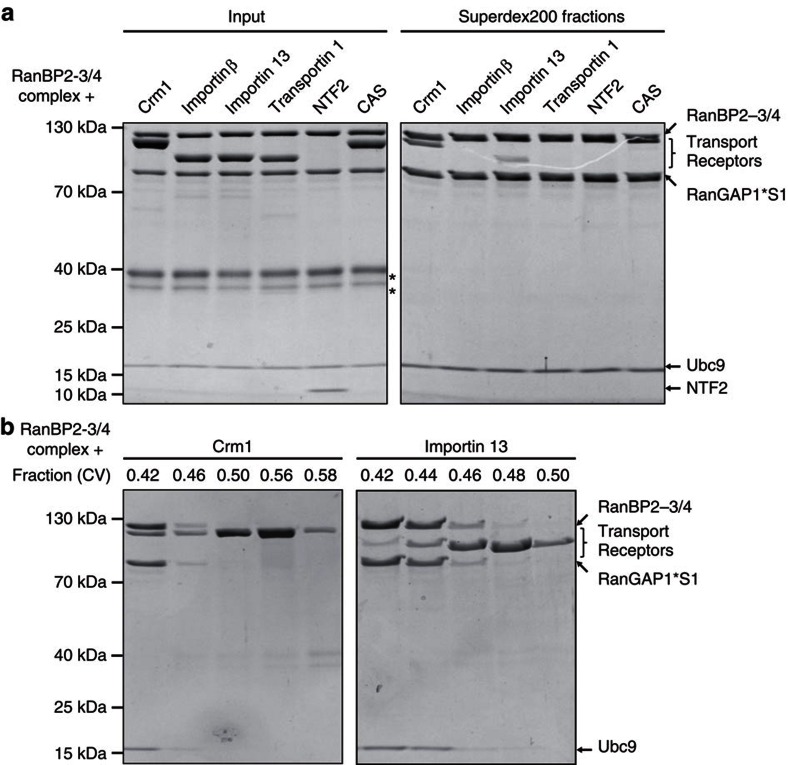
The RanBP2-/4 complex specifically interacts with Crm1. (**a**) Interaction of transport receptors with the RanBP2-3/4 complex in gel filtration. The RanBP2-3/4 complex (1 μM) was incubated with the indicated transport receptors (2 μM each). Proteins were separated over a Superdex200 5/150 GL gel filtration column. Samples of the input (10%) and of fractions collected at 0.42 CV (column volumes) were analysed by SDS-PAGE and Coomassie staining. *Ovalbumin from buffer; S1, SUMO1. (**b**) Crm1 co-migrates with the RanBP2-3/4 complex in gel filtration, whereas Importin 13 does not; the elution peaks of Importin 13 and the RanBP2-3/4 complex partially overlap. Gel filtration binding assays were performed as described in **a** with the RanBP2-3/4 complex, Crm1 (left) and Importin 13 (right). Fractions collected at 0.42–0.50 CV were analysed by SDS-PAGE and Coomassie staining.

**Figure 3:**
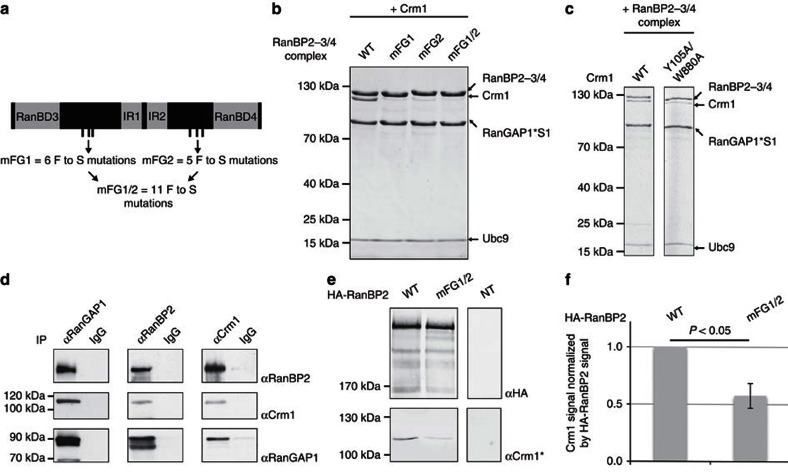
The Crm1/RanBP2 interaction depends on both FG-repeat patches in RanBP2-3/4. (**a**) Scheme of RanBP2-3/4 variants in which the two IR-flanking FG-repeat patches were rendered non-functional for transport receptor interaction by F-to-S mutations^41^ of each patch (6 mutations for the N-terminal patch=mFG1 and 5 mutations for the C-terminal patch=mFG2 of the E3 ligase region). (**b**) Crm1 requires both IR-adjacent FG-repeat patches of RanBP2-3/4 for a stable interaction. RanBP2-3/4 complex variants (wild-type and FG-mutants as depicted in **a**—1 μM each) were incubated with Crm1 (2 μM) and separated over a Superdex200 5/150 GL gel filtration column. Samples were collected at 0.42 CV and analysed by SDS-PAGE and Coomassie staining. S1, SUMO1. (**c**) Mutations in conserved FG-binding patches of Crm1 reduce binding to the RanBP2-3/4 complex. The RanBP2-3/4 complex (500 nM) was incubated with Crm1 variants (1 μM) and separated over a Superdex200 5/150 GL gel filtration column. Samples were collected at 0.44 CV and analysed by SDS-PAGE and Coomassie staining. (**d**) Crm1 associates with the endogenous mitotic RanBP2 complex. Immunoprecipitations with the indicated antibodies were performed from nocodazole-arrested HeLa CSH cells. Immunoprecipitates were analysed by αRanBP2 (upper panels), αCrm1 (middle panels) and αRanGAP1 immunoblots (lower panels). (**e**,**f**) The FG-repeats flanking the IR-region significantly contribute to the association of Crm1 with the mitotic RanBP2 complex. HEK293T cells were depleted of endogenous RanBP2 by double siRNA treatment and transfected with siRNA resistant, full-length, HA-tagged RanBP2 constructs: wild-type (WT) or a variant lacking the E3 ligase-adjacent FG-repeats (mFG1/2). After nocodazole arrest, cells were lysed, subjected to αHA immunoprecipitation and the amount of co-immunoprecipitated Crm1 was analysed by SDS-PAGE and immunoblotting. As a control, αHA immunoprecipitation was performed from cells that were treated with non-targeting siRNA (NT). Representative αHA (top) and αCrm1* (bottom) immunoblots from αHA immunoprecipitation are shown in **e** and the quantification of Crm1 signals normalized to the HA-RanBP2 signals from three biological replicates are shown in **f**. Error bars=s.e.m.; significance was calculated by one-sided paired Student's *t*-test (*P*=0.029); αCrm1*=self-made Crm1 antibody.

Next, we wanted to assess whether the interaction between RanBP2-3/4 and Crm1 observed *in vitro* also takes place in cells. We had previously shown that the endogenous RanBP2 complex can be immunoprecipitated from prometaphase HeLa cytosol^44^. As shown in Fig. 3d, Crm1 co-enriched with the endogenous RanBP2 complex, which is indicative of a high-affinity, stable interaction. However, full-length RanBP2 has many FG-repeats (see Fig. 1) and also harbours a Crm1-binding site in its zinc-finger cluster^45^. To test the specific contribution of the two FG-repeat patches flanking the IR-region, we knocked-down endogenous RanBP2 in HEK293T cells by siRNA and re-transfected siRNA-resistant HA-tagged full-length versions of RanBP2. On prometaphase arrest, cell lysis and αHA immunoprecipitation, we compared the levels of co-precipitating Crm1 (Fig. 3e). Indeed, mutating the two FG-repeat patches in the RanBP2-3/4 region of full-length RanBP2 significantly reduced its interaction with Crm1 by about 40% (Fig. 3f). Whereas residual Crm1 co-immunoprecipitation is perfectly in line with additional binding sites, the clear reduction in Crm1 levels underlines the importance of the two FG-patches in the RanBP2-3/4 region of the RanBP2 complex in cells.

### Ran strengthens the interaction between RanBP2-3/4 and Crm1

**Figure 4:**
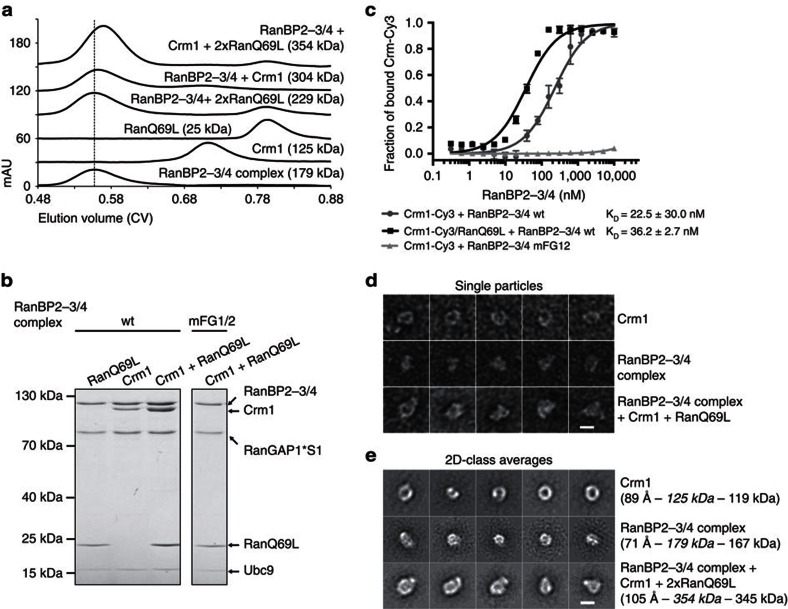
Binding of Crm1 and RanQ69L reduces the structural flexibility of the RanBP2-3/4 complex. (**a**) On Crm1 and RanQ69L binding, the Stokes radius of the RanBP2-3/4 complex does not increase. RanBP2-3/4 complex variants (1 μM each), Crm1 (1.5 μM) and RanQ69L (3 μM) were incubated as indicated and separated over Superose6 10/300 GL gel filtration column. Displayed are chromatograms recorded at 280 nm. The molecular weights (calculated in italics and determined by SEC-MALS in bold; Supplementary Fig. 1d) of analysed proteins and protein complexes are indicated. For molecular weight calculations, we assumed a binding stoichiometry between RanQ69L and RanBP2-3/4 of 2:1. Dashed line=elution volume of the RanBP2-3/4 complex. (**b**) In the presence of RanQ69L, Crm1 binds more strongly to the RanBP2-3/4 complex. Gel filtration binding assays were performed as in **a**. Samples collected at 0.56 CV were analysed by SDS-PAGE and Coomassie staining. S1, SUMO1. (**c**) Dissociation constant of the interaction between RanBP2-3/4 and Crm1 determined by MST. Cy3-labelled Crm1 (20 nM) was incubated with RanBP2-3/4 fragments (0.3 nM—10 μM) in the absence and presence of RanQ69L (30 μM) and subjected to MST (laser-power 70%, LED-power 20%). Shown is a summary of three biological replicates per sample with values normalized to the fraction of bound Crm1-Cy3. Error bars=s.e.m. (**d**) Electron micrographs of uranyl acetate-stained Crm1, RanBP2-3/4 complex and RanBP2-3/4 complex bound to Crm1 and RanQ69L. Images were obtained at 120 keV; representative particles are shown. Crm1 (top row) was purified and imaged directly. The RanBP2-3/4 complex alone (middle row) and bound to Crm1 and RanQ69L (bottom row) were cross-linked with glutaraldehyde, purified and then imaged. Scale bar, 10 nm. (**e**) Selected 2D class averages of the particles from Crm1, the RanBP2-3/4 complex alone and bound to Crm1 and RanQ69L from **d** are shown. The average diameter along the longest axis of the analysed particles and the molecular weights are indicated (calculated molecular weights in italics and determined by SEC-MALS in bold; see Supplementary Fig. 1d). Scale bar, 10 nm.

In light of two high-affinity RanBDs in RanBP2-3/4, we next addressed whether Ran-GTP influences the interaction between Crm1 and the RanBP2-3/4 complex. To prevent Ran-GTP hydrolysis by the complex component RanGAP1, we used the hydrolysis-deficient RanQ69L mutant^46^. As shown in Fig. 4a,b, RanQ69L not only co-migrated with the RanBP2-3/4 complex in gel filtration, it also increased the amount of co-migrating Crm1 to stoichiometric levels. However, the interaction of Crm1 with the RanBP2-3/4 complex remained strictly dependent on the FG-repeat patches in the presence of RanQ69L. Thus, the increase in Crm1 co-migration seems to depend on cooperative interactions. Consistent with this observation, microscale thermophoresis (MST) experiments with Cy3-labelled Crm1 and the RanBP2-3/4 fragment revealed an apparent K_D_ of about 230 nM in the absence—and of 36 nM in the presence of an excess of RanQ69L (Fig. 4c).

### Binding to Crm1 restricts the flexibility of RanBP2-3/4

The region between the two FG-repeat patches in the RanBP2-3/4 fragment (about 300 aa) was shown to be natively unfolded^19^. The requirement of both FG-repeat patches for Crm1-binding implies that (1) both FG-repeat patches serve as anchoring points for RanBP2 and (2) that this anchoring somewhat restricts the degrees of freedom of the intervening sequence region. Consistent with this idea was the qualitative behaviour of RanBP2-3/4 complexes on gel filtration: binding of 125 kDa Crm1 did not substantially increase the Stokes radius of the 178 kDa RanBP2-3/4 complex (Fig. 4a for Superose 6 and Supplementary Fig. 1 for Superdex 200). This effect was also observed, when we used a smaller complex lacking RanGAP1's catalytic domain (=RanBP2-3/4 Δcat complex) or the RanBP2-3/4 fragment alone rather than a complex (Supplementary Fig. 1b). Most striking was the effect, when we included RanQ69L: the partition coefficient of the RanBP2-3/4 complex approximated that of a globular protein when bound to Crm1 and RanQ69L (Supplementary Fig. 1c) and its Stokes radius even decreased on a Superose6 column (Fig. 4a) despite a doubling of the calculated molecular mass (assuming a binding of one RanQ69L molecule on each RanBD of RanBP2-3/4). Of note, to ensure that the peak fractions in gel filtration contained the expected components, we turned to SEC-MALS: The molecular masses of the RanBP2-3/4 complex, Crm1 and the RanBP2-3/4 complex bound to Crm1 and two molecules of RanQ69L agreed with the calculated values (Supplementary Fig. 1d).

To gain further evidence for the apparent restriction of flexibility of RanBP2, we turned to electron microscopy (EM) analysis (Fig. 4d,e and Supplementary Fig. 2). Micrographs of purified Crm1 showed the expected toroid-like shape (Fig. 4d,e)^47^,^48^,^49^. In micrographs of the negatively stained and cross-linked RanBP2-3/4 complex, we observed irregular particles, which appeared smaller than Crm1 particles (Fig. 4d,e and Supplementary Fig. 2c). The unstructured parts of the complex (for example, regions between RanBD3 and IR1 as well as IR2 and RanBD4) would be hard to see in negative staining and it is possible that the visible part only corresponds to a globular portion of the complex (IR1/RanGAP1*SUMO1/Ubc9=102 kDa—Fig. 4d,e). In contrast, micrographs of a purified and cross-linked RanBP2-3/4 complex bound to Crm1 and RanQ69L showed homogeneous particles (Fig. 4d,e). The average diameter of these particles appeared larger than the ones of Crm1 alone. Taken altogether, these findings are compatible with a steric confinement of the flexible RanBP2-3/4 fragment as it attaches to Crm1 on at least two sites. Moreover, the effect of RanQ69L on the Stokes radius is indicative of additional interactions involving the RanBDs of RanBP2 (see below).

### The RanBP2-3/4 complex interacts with Crm1 export complexes

**Figure 5:**
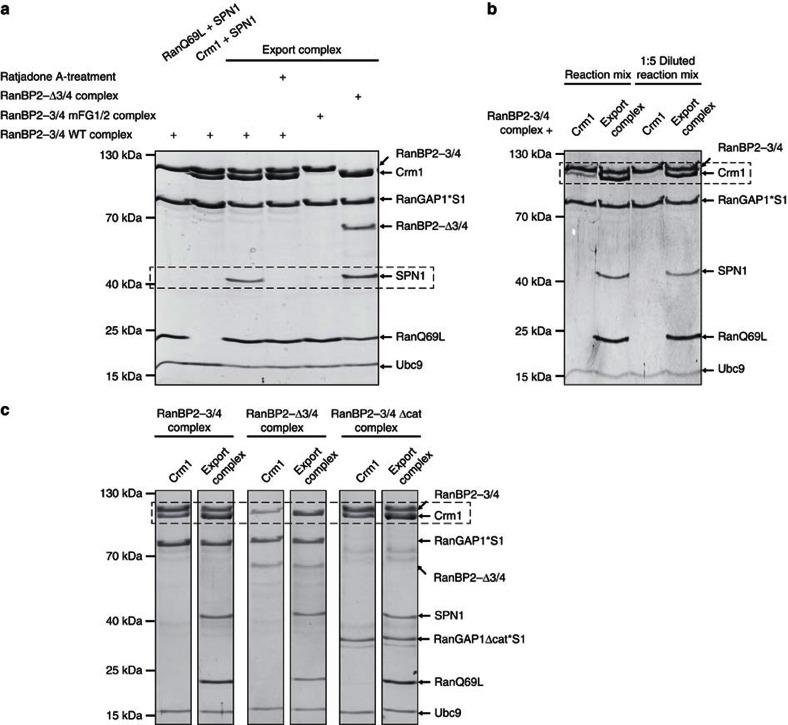
The RanBP2-3/4 complex binds a Crm1 export complex with high affinity via its FG-patches. (**a**) The RanBP2-3/4 complex accommodates a Crm1/RanQ69L/SPN1 export complex in an FG-dependent manner. Ratjadone A or mock treated Crm1 (2 μM each), SPN1 (4 μM) and RanQ69L (5 μM) were pre-incubated before adding RanBP2-3/4 complex variants or the RanBP2-Δ3/4 complex (1 μM each) as indicated (export complex=Crm1+SPN1+RanQ69L). The binding reactions were separated over a Superdex200 5/150 GL gel filtration column. Samples collected at 0.42 CV for the RanBP2-3/4 complex and at 0.46 CV for the RanBP2-Δ3/4 complex were analysed by SDS-PAGE and Coomassie staining. Dashed boxes indicate important regions of the gels. (**b**) The RanBP2-3/4 complex binds to a Crm1/RanQ69L/SPN1 export complex with higher affinity than to Crm1 alone. Binding reactions were set up as in **a** and applied to a Superdex200 5/150 GL gel filtration column either directly (lanes 1 and 2) or after fivefold dilution (lanes 3 and 4—final concentration of 0.2 μM RanBP2-3/4 complex, 0.4 μM Crm1, 0.8 μM SPN1 and 1 μM RanQ69L). Samples were analysed by SDS-PAGE and Coomassie staining. (**c**) Increased affinity of the RanBP2-3/4 complex for the Crm1 export complex does not depend on RanBDs and the catalytic domain of RanGAP1. Crm1 (2 μM), SPN1 (4 μM) and RanQ69L (5 μM) were pre-incubated before adding the RanBP2-3/4 complex, the RanBP2-Δ3/4 complex or the RanBP2-3/4 Δcat complex (1 μM each) as indicated. The binding reactions were separated over a Superdex200 5/150 GL gel filtration column. Samples collected at 0.42 CV for the RanBP2-3/4 complex, at 0.46 CV for the RanBP2-Δ3/4 complex and at 0.44 CV for the RanBP2-3/4 Δcat complex were analysed by SDS-PAGE and Coomassie staining. S1, SUMO1.

Up to this point, we had studied interactions of the RanBP2 complex with the empty Crm1 receptor or with Crm1 and an excess of RanQ69L. To test whether the FG-repeat patches in RanBP2-3/4 can also accommodate proper cargo-containing export complexes, we pre-formed an export complex using 2 μM Crm1, 4 μM of the model NES-cargo Snurportin1 (=SPN1)^50^,^51^ and 5 μM RanQ69L (Supplementary Fig. 3a), and performed binding assays with the RanBP2-3/4 complex (1 μM). Of note, here we used a molar excess of RanQ69L to prevent a possible side reaction: RanBP2's RanBDs may act to dissociate the cargo (see next section). This could be suppressed by saturating RanBDs with free RanQ69L. Indeed, under these conditions, all components of the export complex co-migrated with the RanBP2-3/4 complex (Fig. 5a—lane 3). This depended on the FG-repeat patches, since the mFG1/2 complex variant supported neither Crm1 nor SPN1 co-migration, but was independent of RanBDs, as co-migration was also found with a RanBP2 complex lacking RanBDs (=RanBP2-Δ3/4 complex, Fig. 5a—lane 5 and 6). Of note, co-migration of RanQ69L in the latter case indicated that a proper export complex had been formed. Consistent with this idea, SPN1 co-migration was lost when either Crm1 or RanQ69L was absent in the binding assays (Fig. 5a—lane 1 and 2). Final evidence for proper export complex formation and binding came from experiments with Ratjadone A-treated Crm1. Ratjadone A, like Leptomycin B, specifically and irreversibly modifies the cargo binding-cleft of Crm1 resulting in its inability to form an export complex (Supplementary Fig. 3A)^52^. Ratjadone A-treatment of Crm1 led to a complete loss of SPN1 co-migration with the RanBP2-3/4 complex (Fig. 5a—lane 4). Taken altogether, these experiments reveal that the RanBP2 complex can bind Crm1 export complexes with a typically sized cargo protein in an FG-dependent manner.

Finally, when we directly compared levels of co-migrating free Crm1 with export complex-bound Crm1, we noticed significantly better binding of the latter (Fig. 5b—lane 1 and 2). Similar observations were made, when we used RanBP2 complexes lacking either the RanBDs or the catalytic domain of RanGAP1 (Fig. 5c), excluding unknown contributions of these components. When we repeated the gel filtration binding assays at significantly lower concentrations (that is, with 0.2 μM RanBP2-3/4 complex, 0.4 μM Crm1, 0.8 μM SPN1 and 1 μM RanQ69L), free Crm1 failed to co-migrate, whereas the Crm1/RanQ69L/SPN1 complex still bound stably to the RanBP2-3/4 complex (Fig. 5b—lane 3 and 4). Consistent with this observation, MST measurements with RanBP2-Δ3/4 and a pre-formed export complex yielded a *K*_D_ of 27 nM (Supplementary Fig. 3B), which is significantly lower than that of RanBP2-3/4 with Crm1. Taken altogether, these findings reveal that Crm1 has a much higher affinity for the RanBP2 complex when locked in an export complex than when alone.

### The RanBP2-3/4 complex can dissociate NES-cargoes from Crm1

**Figure 6:**
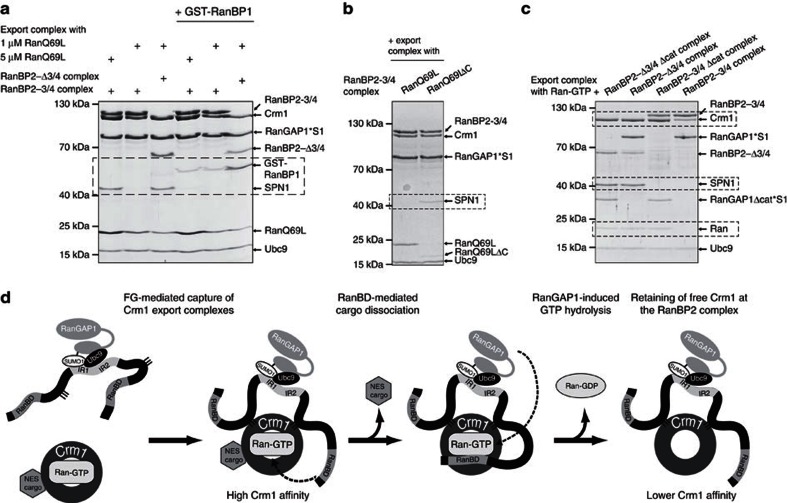
The RanBP2-3/4 complex can disassemble a Crm1-dependent export complex. (**a**) The RanBDs of the RanBP2-3/4 complex dissociate cargo from a Crm1 export complex. Crm1 (2 μM), SPN1 (4 μM) and RanQ69L (1 or 5 μM) were pre-incubated before adding the RanBP2-3/4 complex or the RanBP2-Δ3/4 complex (1 μM each) in the presence or absence of GST-RanBP1 (10 μM) as indicated (export complex=Crm1+SPN1+RanQ69L). Binding reactions were separated over a Superdex200 5/150 GL gel filtration column, and samples collected at 0.42 CV for the RanBP2-3/4 complex and at 0.46 CV for the RanBP2-Δ3/4 complex were analysed by SDS-PAGE and Coomassie staining. S1=SUMO1. Dashed boxes indicate important regions of the gels. (**b**) The RanBP2-3/4 complex does not dissociate cargo from an export complex formed with a RanBD-binding deficient Ran. Binding assays were performed as in **a**. The export complex with RanQ69LΔC was formed by incubating Crm1 (10 μM), SPN1 (30 μM) and RanQ69LΔC (50 μM) and purified by gel filtration. For the binding assay, 1 μM purified export complex was used. (**c**) The RanBDs and RanGAP1*SUMO1 in the RanBP2-3/4 complex can disassemble the Crm1 export complex. Binding assays were performed as in **a**. RanBP2 complex variants built with RanBP2-3/4 or RanBP2-Δ3/4 and RanGAP1*SUMO1 or RanGAP1Δcat*SUMO1 were added as indicated. Samples were collected at 0.42 CV for the RanBP2-3/4 complex, at 0.44 CV for the RanBP2-3/4 Δcat complex, at 0.46 CV for the RanBP2-Δ3/4 complex and at 0.48 CV for the RanBP2-Δ3/4 Δcat complex. (**d**) Model of the RanBP2 complex as an autonomous disassembly platform for Crm1 export complexes. From left to right: the partly unfolded RanBP2 complex binds a Crm1 export complex with high affinity with its two IR-adjacent FG-patches thus restricting RanBP2's flexibility. One of the RanBDs of RanBP2 engages the export complex (dashed arrow) resulting in cargo-dissociation and formation of a Crm1/Ran-GTP/RanBD sub-complex. Following its deprotection in the export complex, Ran-GTP is engaged by the catalytic domain of RanGAP1 (dashed arrow). GTP-hydrolysis is induced and Ran-GDP diffuses away leaving behind less stably bound Crm1. The last three stages of this model of Crm1 export complex disassembly were reconstituted *in vitro* (see text).

The finding that the RanBP2-3/4 complex can stably interact with Crm1 export complexes prompted the question whether it can also disassemble them. Canonical export complex disassembly starts with binding of a RanBD to Ran-GTP embedded in the export complex. Formation of a Crm1/Ran-GTP/RanBD complex and allosteric changes in Crm1 result in the dissociation of the NES-cargo^6^,^8^. It has previously been shown that isolated RanBDs of RanBP2, like RanBP1, can function in this way^6^. To assess whether they also work when part of the RanBP2 complex, we tested export complex co-migration with the RanBP2-3/4 complex under two experimental conditions: (a) as above with an excess of RanQ69L to saturate RanBDs (5 μM RanQ69L, 2 μM Crm1, 4 μM SPN1 and 1 μM RanBP2-3/4 complex, that is, 2 μM RanBDs) and (b) with limiting amounts of RanQ69L (as above, but with 1 μM RanQ69L). As expected, SPN1 co-migrated with the RanBP2-3/4 complex in the presence of an excess of RanQ69L (Fig. 6a—lane 1). In contrast, SPN1 was entirely lost when RanQ69L was limiting (Fig. 6a—lane 2). This seemed to require the RanBDs, because SPN1 was not lost when the RanBP2-Δ3/4 complex variant was used (Fig. 6a—lane 3). To gain further evidence for a role of the RanBDs in export complex disassembly, we turned to a Ran variant lacking the C-terminal 36 aa, RanQ69LΔC. This Ran variant forms an export complex but cannot engage in a functional Ran/RanBD interaction and thus does not allow RanBD-mediated cargo-dissociation from export complexes^6^,^51^. Indeed, an export complex pre-formed with RanQ69LΔC was not dissociated in the presence of the RanBP2-3/4 complex (Fig. 6b). Taken altogether, these findings reveal that the RanBDs in the RanBP2-3/4 complex are able to promote NES-cargo-dissociation from a Crm1 export complex.

Use of the hydrolysis-deficient RanQ69L mutant in these experiment should trap intermediate interactions during export complex disassembly, that is, allow formation of a stable Crm1/RanQ69L/RanBD intermediate^6^. Consistent with this idea were findings with soluble GST-RanBP1 (Fig. 6a): when included in binding assays, GST-RanBP1 dissociated SPN1 and co-migrated with the remaining components (that is, RanBP2-Δ3/4 complex, RanQ69L and Crm1, Fig. 6a—lane 6). Intriguingly, comparably little GST-RanBP1 co-migrated with RanBP2-3/4 complexes, even though it was offered in 10-fold molar excess (Fig. 6a—lanes 3 and 4). This indicated that RanBDs available in *cis* (from the RanBP2-3/4 complex) are more effective in engaging in the Crm1/RanQ69L/RanBD interaction than those added in *trans* (from GST-RanBP1). From these findings we concluded that cargo-dissociation in the RanBP2-3/4 complex takes place in *cis*. This is marked by the formation of a stable Crm1/RanQ69L/RanBD sub-complex within a RanBP2-3/4 complex, in which Crm1 remains anchored to RanBP2-3/4 via the FG-repeat patches and via a RanBD-tethered RanQ69L. This sub-complex formation also explains the positive effect that RanQ69L has on the affinity of Crm1 for the RanBP2-3/4 complex (see Fig. 4a).

### The RanBP2-3/4 complex can promote Ran-GTP hydrolysis

To test whether the RanBP2 complex is sufficient to also catalyze the last step of export complex disassembly, that is, Ran-GTP hydrolysis, we repeated interaction studies with export complexes that contained GTP-loaded wild-type Ran rather than the hydrolysis-deficient RanQ69L mutant (Fig. 6c). First, we tested a RanBP2 complex variant lacking both RanBDs and the catalytic domain of RanGAP1 (RanBP2-Δ3/4 Δcat complex). As expected, this complex supported stable binding of the export complex, which was revealed by co-migration of Crm1, SPN1 and Ran (Fig. 6c—lane 1). We interpret this species as the first intermediate in export complex disassembly at the RanBP2 complex (Fig. 6d—second from the left). A complex without RanBDs but with full-length RanGAP1 (RanBP2-Δ3/4 complex) also allowed co-migration of SPN1 and Ran, which underlined the protection of Ran-GTP from RanGAP1 activity in the context of a RanBP2-associated export complex (Fig. 6c—lane 2). When we used a RanBP2 complex with RanBDs but without the catalytic domain of RanGAP1 (RanBP2-3/4 Δcat complex), co-migration of the cargo, that is, SPN1, was lost, whereas Ran co-migration was preserved (Fig. 6c—lane 3). Altogether with the data shown in Fig. 4, we interpreted this species as an intermediate step in export complex disassembly marked by the formation of the Crm1/Ran-GTP/RanBD sub-complex (Fig. 6d—second from the right). Finally, we tested co-migration of the export complex with a RanBP2 complex that contained both RanBDs and RanGAP1 (RanBP2-3/4 complex). Intriguingly, neither SPN1 nor Ran co-migrated with the complex, in contrast to experiments in Fig. 6a, where RanQ69L remained bound (Fig. 6c—lane 4). These findings indicate that the RanBP2-3/4 complex is able to hydrolyze Ran-GTP on cargo displacement. In the resulting species, Crm1 is bound to the RanBP2-3/4 complex. It represents the last intermediate of nuclear export complex disassembly, which may also be the first intermediate for Crm1's way back into the nucleus (Fig. 6d—right). Altogether, these experiments demonstrate that the RanBP2 complex can indeed function as an autonomous disassembly platform for Crm1-containing export complexes.

### Crm1 binding is compatible with SUMO E3 ligase function

**Figure 7:**
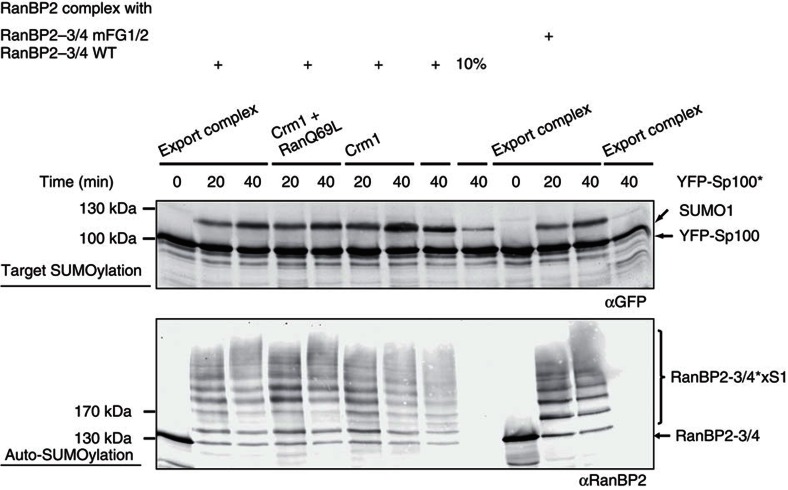
Export complex binding is compatible with the E3 ligase activity of the RanBP2-3/4 complex. Crm1, RanQ69L and SPN1 (2.5 μM each) were pre-incubated (export complex (Crm1+SPN1+RanQ69L)), added to RanBP2-3/4 complex variants (25 nM) as indicated, and the mixture was used as E3 ligase in *in vitro* SUMOylation reactions containing YFP-Sp100 (500 nM), SUMO E1 enzyme (100 nM), Ubc9 (50 nM) and SUMO1 (10 μM). 10%=the reaction was performed with 2.5 nM RanBP2-3/4 wild-type complex in the absence of export complex components. YFP-Sp100 SUMOylation was analysed by immunoblotting with αGFP (top). Auto-SUMOylation of RanBP2-3/4 was analysed by immunoblotting with αRanBP2 (bottom). RanBP2-3/4*xS1, polySUMOylated RanBP2-3/4.

As described above, export complex-binding to the RanBP2 complex takes place in an area that also contains RanBP2's SUMO E3 ligase region. Hence, we asked whether Crm1- or export complex-binding would impinge on RanBP2's SUMO E3 ligase activity. For this, we reconstituted the three stable intermediates of export complex disassembly at the RanBP2 complex: (1) RanBP2-3/4 complex bound to a Crm1/RanQ69L/SPN1 export complex, (2) RanBP2-3/4 complex bound to Crm1 and RanQ69L and (3) RanBP2-3/4 complex bound to Crm1 alone (Fig. 6d). The SUMO E3 ligase activity of these complexes was compared with that of the RanBP2-3/4 mFG1/2 complex, which cannot bind Crm1. Two criteria were used to assess activity: SUMOylation of the model substrate YFP-Sp100 and auto-SUMOylation of RanBP2-3/4 (Fig. 7). To allow complex saturation even at the low E3 concentration needed for *in vitro* SUMOylation (25 nM), we added Crm1, RanQ69L and SPN1 in 100-fold molar excess. More than 90% of the RanBP2-3/4 complex should be occupied by Crm1 under these conditions (K_D_ of 230 nM, Fig. 4c). To determine the maximal contribution of free complex, we also measured activity of a SUMOylation reaction with 2.5 nM RanBP2-3/4 complex. As shown in Fig. 7, all complexes were active as SUMO E3 ligases, both with respect to YFP-Sp100 SUMOylation and in auto-SUMOylation. No significant differences could be observed to the activity of 25 nM free RanBP2-3/4 complex, and all complexes showed significantly higher activity than 2.5 nM RanBP2-3/4 complex. We thus conclude that Crm1, Crm1/RanQ69L and Crm1/RanQ69L/SPN1 have no deleterious effects on RanBP2's SUMOylation activity *in vitro*. In consequence, the RanBP2 complex is an active SUMO E3 ligase even when associated with nuclear export complexes.

## Discussion

Here we show that the vertebrate RanBP2 complex, which is an NPC component in interphase and a soluble entity in mitosis, is an autonomous disassembly machine for prototypic Crm1-dependent export complexes (Fig. 6d). This function depends on RanGAP1 and the RanBDs of the complex and is endowed with specificity by two FG-repeat patches in RanBP2, which act as a remarkably stable binding site for Crm1. Transport receptor interactions with FG-repeat containing protein fragments are typically of low affinity and are usually measured with immobilized FG-repeats^39^,^40^. Consistent with this, five of the six transport receptors tested here failed to interact with the RanBP2 complex in a stringent in-solution assay (co-migration in gel filtration), Crm1 being the striking exception. With a *K*_D_ of 230 nM (Fig. 4c), it has a remarkably high affinity for RanBP2. Our finding that two patches of FG-repeats are required for this interaction both *in vitro* and in cells suggested that Crm1, but not other transport receptors, features two distinct FG-repeat binding sites for simultaneous interaction. Two recent studies on Crm1 interactions with FG-repeat-containing proteins provide structural insights into possible interactions with RanBP2. First, Koyama and co-workers^42^ reported on the crystal structure of the *S.cerevisiae* Xpo1p/Gsp1p-GTP/Yrb2p complex (homologue to the mammalian Crm1/Ran-GTP/RanBP3 complex). The predominantly nuclear protein Yrb2, which contributes to nuclear export complex assembly, contains FG-repeat patches and a low-affinity Gsp1p-binding domain (RanBD). As revealed by the structure, Yrb2p's FG-repeat patches interact with two conserved binding pockets on the outer surface of Xpo1p, one at its N- and one at its C-terminus. Second, Port and colleagues^43^ solved a crystal structure of the human Crm1/Ran-GTP/SPN1 export complex bound to a fragment of the FG-nucleoporin Nup214. Their work revealed that Nup214 contacts homologous FG-binding pockets at the human Crm1 termini. Their work also revealed an additional FG-binding pocket located in the C-terminal portion of the export receptor^43^. Our initial mutational analysis of Crm1 suggests that RanBP2 exploits similar N- and C-terminal-binding pockets as Yrb2p and Nup214 (Fig. 3c). While comprehensive understanding of the interaction between Crm1 and RanBP2 will require high-resolution structural analyses, this finding is in line with observations that several FG-containing proteins can compete for Crm1-binding^42^,^43^.

There are two aspects, however, that set the binding of the RanBP2 complex to a Crm1 export complex apart from that of Nup214 and Yrb2p: (1) Yrb2p and Nup214 have stabilizing effects on Crm1 export complexes, whereas the RanBP2 complex is ultimately able to disassemble them (see also below) and (2) whereas the FG-repeat patches of Yrb2p and Nup214 are separated by roughly 30–40 aa, ∼300 aa separate the FG-repeat patches in RanBP2; these include the binding site for RanGAP1*SUMO1 as well as RanBP2s E3 ligase region (discussed below). This extended sequence in RanBP2 may be needed to keep the Ran GTPase-activating protein close to the export complex, while simultaneously allowing positional flexibility for RanGAP1's catalytic domain to reach Ran-GTP in the export complex.

Crm1 is composed of an array of 21 HEAT repeats^47^,^48^,^49^. A slight tilt between consecutive HEAT repeats and their tandem-stacking results in the superhelical, extended shape of free Crm1, which is typical for members of the importin β-superfamily. When bound to Ran and/or NES-cargo, Crm1 assumes a more compacted conformation in which the N- and C-terminus of the molecule come closer altogether; this gives Crm1 a toroid-like shape. Two of the three RanBP2 complexes that we reconstituted should bind Crm1 in its compacted toroid-shape: the complex involving Crm1 and RanQ69L, and the complex binding the Crm1/RanQ69L/SPN1 export complex^6^,^51^. In contrast, Crm1 alone should largely assume the extended conformation^53^. The greater distance of the N- and C-termini of Crm1 in its extended, free state may offer a molecular explanation for its lower affinity to RanBP2-3/4 compared with the compacted conformation of Crm1, for example, in the export complex. This notion is particularly interesting in light of the fact that Nup214 may exert its stabilizing effect on export complexes by keeping Crm1 in the toroid-conformation associated with Ran- and cargo-binding^43^. Similarly, Yrb2p-binding to Xpo1 (and to Gsp1p-GTP), which primes the transport receptor for loading with NES-cargo, also locks it in the closed conformation^42^. Physiologically, the change in affinity in RanBP2 is very plausible, considering that the export complex is the substrate of a disassembly reaction mediated by the RanBP2 complex, whereas free Crm1 is the product.

Disassembly of Crm1 export complexes requires soluble RanGAP1 and RanBP1 in yeast. Here, we demonstrated that vertebrate cells offer a second option, disassembly by the RanBP2 complex. This raises the question whether both possibilities are used as redundant alternatives that make the process more efficient, or whether disassembly by the RanBP2 complex has also specific, non-redundant functions. At least two advantages are conceivable for disassembly by the RanBP2 complex. First, a two-component interaction is sufficient for disassembly by the RanBP2 complex, whereas a three-component interaction is required with soluble RanGAP1 and RanBP1 (RanGAP1 and RanBP1 do not interact with each other). This should make the RanBP2-dependent reaction more efficient, both in interphase and in mitosis. Second, Crm1 remains bound to the RanBP2 complex after export complex disassembly and is hence retained at the NPC during interphase to relocate more quickly into the nucleus. This may be a particular advantage in large cells, in which diffusion may become limiting. Currently available data regarding a requirement of RanBP2 for Crm1-dependent export have led to somewhat conflicting results^28^,^54^,^55^,^56^, as effects on export were either moderate or not detectable. These studies all involved rapidly dividing tissue culture cells that have high levels of soluble RanGAP1 and RanBP1 and are relatively small in size. Altogether, they reveal that soluble RanGAP1 and RanBP1 are sufficient to disassemble export complexes under normal growth conditions. However, RanBP2 may well be needed for export complex disassembly in differentiated cells that have extended dimensions (for example, neurons) and/or with low levels of soluble RanGAP1 or RanBP1. Significant reduction of RanGAP1 levels on differentiation has been described for example, in human coronary artery smooth muscle cells^57^. Finally, disassembly by the RanBP2 complex may not primarily serve to increase efficiency of a general pathway, but may be used to couple export complex disassembly with SUMOylation.

The structural overlap of RanBP2's nuclear export disassembly and E3 ligase activity strongly suggests that these activities are functionally linked. Consistent with this idea, we showed that the RanBP2 complex remains active as a SUMO E3 ligase when associated with Crm1 in three distinct configurations. Crm1 interacts with a plethora of NES-proteins in the context of export complexes. As shown here, it can bring such proteins into proximity of the E3 ligase region and may thus serve as a substrate adaptor for SUMOylation. We assume that this will only be true for a defined set of Crm1 cargoes, as they need to provide appropriate and accessible lysines. This Crm1-dependent recruitment mechanism may be used during interphase to SUMOylate proteins on their way to the cytoplasm, but may also be relevant during mitosis. In recent years, nuclear transport factors have emerged as key players during vertebrate mitosis^5^. This includes Crm1, which has been implicated in recruiting the RanBP2 complex to kinetochores in an export complex-dependent manner^58^ and has been shown to be crucial for stable kinetochore/microtubule attachment^29^,^59^. The Crm1/RanBP2 interaction described here may offer a molecular explanation for this recruitment, if Crm1 forms an export complex with an NES-protein at the kinetochore. In light of the finding that many kinetochore proteins are subject to SUMOylation^60^, this is an attractive model for further investigations.

In conclusion, we described a novel stable interaction between RanBP2 and Crm1, which renders the RanBP2 complex a molecular entity to autonomously bind and disassemble Crm1 export complexes. This interaction, which frames the SUMO E3 ligase region of RanBP2, is compatible with E3 ligase activity and may thus point to an exciting link between SUMOylation and export complex disassembly.

## Methods

### Expression constructs

Bacterial expression constructs for Ubc9, SUMO E1 enzyme, SUMO1, RanBP2-3/4-His (aa 2,304–3,062), RanGAP1, His-RanGAP1Δcat (aa 398–587), Ran and His-YFP-Sp100 have been described previously^17^,^19^,^30^,^60^,^61^. Bacterial expression plasmids pQE30-His-CAS, pQE32-His-Transportin1, pQE60-Crm1-His, pET3-RanQ69L, pQE80-Importin13-His and pET30a-Importinβ-His were kindly provided by Ralph Kehlenbach (Georg-August University of Göttingen); pET30a-Importinβ-His was recloned into pET23a using BamHI and NotI. pET-NTF2 was a kind gift of Dirk Görlich (Max-Planck-Institute for Biophysical Chemistry, Göttingen) and RanQ69LΔC (aa 1–180) from Thomas Monecke and Dirk Görlich^51^.

RanBP2-3/4 mFG variants and the Crm1-Y105A/W880A mutant^42^ were obtained by (multi)site-directed mutagenesis (Agilent). For RanBP2-Δ3/4, a PreScission and TEV cleavage site were introduced into the RanBP2-3/4 construct by insertion mutagenesis^62^ (PreScission site before amino acid 2,474 after RanBD3 and TEV site after amino acid 2,887 before RanBD4).

The mammalian expression construct for full-length wild-type HA-RanBP2 (pEF-HA-RanBP2) has been described previously^63^ and was kindly provided by Sarah Wälde (Department of Cell Biology, Yale School of Medicine). The HA-RanBP2 mFG1/2 variant was obtained by cloning the coding region of pET23a-RanBP2-3/4-His mFG1/2 into pEF-HA-RanBP2 construct by DraIII and SexAI. Human RanBP1 amplified by PCR was cloned into pGEX-6-P3 by BamHI and XhoI.

### Primers

BP2-FG1a 5′- GAAAAATCAAAACCATCTGCATCCGGCAACAGTTCAG -3′,

BP2-FG1b 5′- GAAAAATCAAAACCATCTGCATCCGGCAACAGTTCAG -3′,

BP2-FG1c 5′- GGGTCTTTGTCTGGATCTAGTTTTAATGCACC -3′,

BP2-FG2a 5′- AAGATAGTTTCATCTGGATCTGGAAGTAGCACAGGG -3′,

BP2-FG2b 5′- TCCAGTAATTCTGGAGATTCTGCTTCTGGTTCTAAG -3′,

BP2-FG2c 5′- ACTGGAGCAGCTGTGTCTGGAACACAGTCAGTCGG -3′,

BP2-PreSci forward 5′- TGTAGCTGTATTAGAAGTACTATTCCAG GGTCCAGAAACCACAAGAGAGAGG -3′,

BP2-PreSci reverse 5′- TACTTCTAATACAGCTACAGCAATTTTGCCACATGGTGACTCTCTGGG -3′,

BP2-TEV forward 5′- TCAGTCGGAACCGAGAATCTGTACTTCCAGTCAGCCGGTAAAGTTGGTGAAG -3′,

BP2-TEV reverse 5′- TCGGTTCCGACTGACTGTGTTCCAAACACAGCTGCTCCAGTATTTGC -3′,

Crm1-Y105A forward 5′- GTGCGAAGGAATAAAAAAAGCCGTTGTTGGCCTCATTATCAAG -3′,

Crm1-Y105A reverse 5′- CTTGATAATGAGGCCAACAACGGCTTTTTTTATTCCTTCGCAC -3′,

Crm1-W880A forward 5′- CTTGTTTTGGATTCCATCATTGCGGCTTTCAAACATACTATGAGG -3′,

Crm1-W880A reverse 5′- CCTCATAGTATGTTTGAAAGCCGCAATGATGGAATCCAAAACAAG -3′.

### Antibodies

Mouse αRan was from BD Transduction Laboratories (1:2,000, catalogue number: 610341); mouse αHA.11 (1:500, catalogue number: MMS-101P) was from Sigma; rabbit αGFP (1:1,000, catalogue number: sc-8334), rabbit αUbc9 (1:1,000, catalogue number: sc-10759), goat αCrm1 (1:1,000, catalogue number: sc-7826; used for immunoprecipitation experiments) and goat αSPN1 (1:1,000, catalogue number: sc-55301) were from Santa Cruz. Affinity purified goat αRanBP2 (1:1,000) and goat αRanGAP1 (1:1,000) have been described elsewhere^16^,^29^. Goat αCrm1* (1:1,000 from a 0.2 mg ml^−1^ stock) was affinity purified from goat αCrm1 serum (raised against the C-terminally His-tagged, full-length Crm1 protein—see above). Uncropped western blots are shown in Supplementary Fig. 4.

### Protein expression and purification

Ubc9, SUMO E1 enzyme, SUMO1, RanBP2-3/4-His, RanGAP1, His-RanGAP1Δcat, His-YFP-Sp100, NTF2, Ran were purified using published procedures^17^,^19^,^30^,^37^,^51^,^64^,^65^. As a final step in purification, all proteins were subjected to gel filtration chromatography in TB (transport buffer: 20 mM HEPES-KOH pH 7.3, 110 mM KAcO, 2 mM Mg(AcO)_2_, 1 mM EGTA, 1 mM DTT and 1 μg ml^−1^ of each aprotinin, leupeptin, pepstatin A). SPN1 was kindly provided by Achim Dickmanns and Ralf Ficner (Georg-August University of Göttingen)^51^.

Quantitative SUMOylation of RanGAP1 and RanGAP1Δcat was performed by incubating 10 μM protein with 30 μM SUMO1, 125 nM Ubc9, 125 nM SUMO E1 enzyme and 5 mM ATP in TB supplemented with 0.05% (v/v) Tween20 at 30 °C for 3 h. For purification, the SUMOylation reaction was concentrated and applied to a Superdex200 10/300 GL column equilibrated in TB^17^.

RanBP2 complex variants were formed by incubating 24 μM Ubc9, 20 μM RanGAP1 variants and 24 μM RanBP2-3/4 variants in TB supplemented with 0.05% (v/v) Tween20 on ice overnight. For purification, complex-forming reactions were applied to a MonoQ 5/50 GL anion exchange column equilibrated in MonoQ buffer A (50 mM Tris-HCl pH 8.0 1 mM DTT and 1 μg ml^−1^ of each aprotinin, leupeptin, pepstatin A), washed with at least 3 CV and eluted by applying a linear gradient of MonoQ buffer B (buffer A supplemented with 1 M NaCl) over 13 CV^17^. For complex variants containing full-length RanGAP1 a gradient of 30–55% of MonoQ buffer B was applied, for variants containing RanGAP1Δcat a gradient of 20–32% was chosen.

For preparative reconstitution of the RanBP2-3/4 complex bound to Crm1 and RanQ69L for EM experiments, complex formation reactions were set up in TB containing 10–20 μM purified RanBP2-3/4 complex, Crm1 in a 1.5-fold molar excess and RanQ69L in a threefold molar excess. The reaction was incubated on ice overnight and then applied to a MonoQ 5/50 GL anion exchange column equilibrated in MonoQ buffer A (without protease inhibitors). Proteins were eluted with a linear gradient from 30–55% of MonoQ buffer B (without protease inhibitors).

RanBP2-Δ3/4 was obtained by expression and purification of the RanBP2-3/4 variant containing PreScission and TEV sites. After cleavage with both proteases (0.75 μg ml^−1^ PreScission and 4 μg ml^−1^ TEV per μM cleavage site) in 50 mM Tris-HCl pH 7.5, 150 mM NaCl and 1 mM DTT, the cleaved RanBDs and the proteases were separated from the RanBP2-Δ3/4 fragment by Superdex200 gel filtration in TB.

For GTP-loading of Ran, 20–100 μM Ran was supplemented with a 25-fold molar excess of GTP over Ran, a 100-fold molar excess of ATP and a 600-fold molar excess of EDTA. After incubation for 30 min at 30 °C, the loading reaction was quenched by the addition of a fivefold molar excess of Mg(AcO)_2_ over EDTA, concentrated using a 5 kDa MWCO concentrator (Vivaspin), and applied to a HiTrap desalting column equilibrated in MonoQ buffer A supplemented with 1 mM MgCl_2_. Protein-containing fractions were pooled and applied to a MonoQ 5/50 GL anion exchange column equilibrated in the same buffer. Proteins were eluted with a linear gradient from 0–30% of MonoQ buffer B supplemented with 1 mM MgCl_2_ in 20 CV, which separated Ran-GDP from Ran-GTP (protocol adapted from ref. 66).

Importinβ-His, Importin13-His, Crm1-His, His-Transportin, His-CAS were purified by Ni-NTA pull-down and Superdex200 gel filtration in TB. His-CAS was additionally purified over MonoQ 5/50 GL column with MonoQ buffer A and B, followed by buffer exchange to TB. GST-RanBP1 was purified by GST pull-down and Superdex200 gel filtration in 50 mM Tris-HCl pH 8.0, 100 mM NaCl, 1 mM DTT and 1 μg ml^−1^ of each aprotinin, leupeptin, pepstatin.

### *In vitro* binding and SUMOylation assays

Binding reactions were set up with the indicated proteins at the indicated concentrations in SAB (SUMOylation assay buffer: TB supplemented with 0.2 mg ml^−1^ ovalbumin and 0.05% (v/v) Tween) and incubated on ice overnight. In all, 50 μl of the binding reactions were applied to the indicated gel filtration columns equilibrated in TB. Peak fractions of 60 μl were collected as indicated and supplemented with 4 × Laemmli buffer and analysed by SDS-PAGE (5–20% gradient gels) and Colloidal Coomassie staining (typically, loading of 35 μl per fraction).

To inhibit Crm1 (refs 52, 67), 6 μM recombinant Crm1 in SAB was incubated with 24 μM Ratjadone A dissolved in methanol for 20 min at room temperature. Ratjadone A and mock treatments (methanol only) were always performed directly before the addition of Crm1 to binding reactions.

Analytical SUMOylation reactions were performed with 500 nM YFP-Sp100, 10 μM SUMO1, 100 nM SUMO E1 enzyme, 50 nM Ubc9 and 2.5 or 25 nM RanBP2-3/4 E3 ligase complex in the absence or presence of 2.5 μM Crm1, RanQ69L and SPN1 in SAB. Reactions were started by the addition of 5 mM ATP and incubated at 30 °C. Reactions were stopped by the addition of 2 × Laemmli buffer and analysed by SDS-PAGE (5–20% gradient gels) and immunoblotting.

### Size exclusion multi-angle light scattering

The molecular weight of the RanBP2-3/4 complex, Crm1 and the RanBP2-3/4 complex bound to Crm1 and RanQ69L was determined by SEC-MALS. A binding reaction (100 μl) was set up with the RanBP2-3/4 complex (4 μM), Crm1 (8 μM) and RanQ69L (20 μM) in TB and incubated for 4 h on ice. In parallel, 100 μl of the RanBP2-3/4 complex (4 μM) and Crm1 (8 μM) were prepared in TB separately. Protein samples (25 μl of each sample) were injected and separated on a Superdex200 Increase PC 2.3/300 GL column (GE Healthcare) equilibrated in TB (without protease inhibitors) with an Äkta Pure (GE Healthcare). Data were collected by Wyatt miniDAWN TREOS and Optilab T-rEX differential refractive index detectors, and analysed with the ASTRA VI software package (Wyatt Technology Corporation). The molecular weight was determined from the Raleigh ratio calculated by measuring the static light scattering and corresponding protein concentration of a selected peak. Yeast alcohol dehydrogenase (150 kDa) and sweet potato β-amylase (200 kDa) served as a calibration standard (not shown).

### Microscale thermophoresis

For MST measurements, recombinant Crm1 was labelled with Cy3-NHS-ester monoreactive dye (Amersham) according to the manufacturer's instructions. Binding reactions were set up with 20 nM Cy3-Crm1 variants, RanBP2-3/4, RanBP2-Δ3/4, RanQ69L and SPN1 at indicated concentrations in SUMOylation assay buffer. Measurements were performed on a Monolith NT.115 using standard treated capillaries with a laser-power of 20–80% and an LED-power of 20–50%; the ‘laser on time' was set to 20–30 s and the ‘final laser off-time' to 5 s. Regions of the thermophoresis curves were analysed 1 s before laser on and 1 s before laser off with the NTAnalysis Software and GraphPad Prism 5. Samples were measured in three biological replicates; for each biological replicate 2–5 technical replicates were measured.

### Cell culture

293T cells were maintained in DMEM supplemented with 10% (v/v) fetal bovine serum, 2 mM L-glutamine and 1% (w/v) of each penicillin and streptomycin in a 5% CO_2_ incubator at 37 °C.

HeLa suspension cells were propagated in Jokliks medium supplemented with 5% (v/v) newborn calf serum, 5% (v/v) fetal bovine serum and 2 mM L-glutamine. The cells were cultured in spinner flasks at 100 r.p.m. in a 37 °C incubator at 3–10 × 10^5^ cells per ml. Exponentially growing cells were arrested in prometaphase by addition of 150 (for 293T cells) and 75 (for HeLa cells) ng ml^−1^ nocodazole to the medium. After 18 h, mitotic cells were collected. RanBP2 knockdown by siRNA transfection (5′- CACAGACAAAGCCGUUGAA -3′) was performed essentially as described in ref. 28. Transfection of full-length HA-RanBP2 constructs was performed as described in ref. 63.

### Immunoprecipitation

Cells were washed three times with PBS at room temperature. The cell pellet was resuspended in TB supplemented with 50 mM NaF, 1 mM Pefa bloc and phosphatase inhibitor cocktail I (Sigma, 1:100 dilution) and lysed by the addition of 1 μl of a 10% digitonin solution per 5 × 10^6^ cells followed by 20 min incubation on ice. Lysates were cleared by three steps of centrifugation at 4 °C: 10 min at 300 × g, 30 min at 25,000 × g, 60 min at 100,000 × g. Immunoprecipitations were performed using αHA-agarose beads (Sigma) or the indicated antibodies and protein G agarose beads (Roche). Immunoprecipitates were allowed to form for 3–4 h rotating gently at 4 °C and washed four times with TB; bound protein was eluted with Laemmli buffer, and analysed by SDS-PAGE and immunoblotting.

### Electron microscopy

For EM grid preparation, purified RanBP2-3/4 complex either alone or bound to Crm1 and RanQ69L (50 μl, 400 nM, freshly purified over MonoQ 5/50 GL column) was incubated with 250 mM glutaraldehyde for 10 min on ice. Cross-linking reactions were quenched by the addition of 1 M Tris-HCl pH 7.4 and incubation for 20 min on ice. Cross-linked complex were purified on a Superdex200 5/150 gel filtration column equilibrated in TB. Crm1 was not cross-linked, but subjected to gel filtration at the same concentration directly before grid preparation. Purified sample (3 μl each) were placed on the grid (5 nm carbon over lacy plastic), washed twice with water and stained with 3% (w/v) uranyl acetate.

Images were acquired with a Zeiss 923 microscope equipped with a field-emission gun and an energy filter operating at 120 keV. Images were acquired using an F416 (4k, TVIPS; Germany) at a pixel size of 1.2 Å/pix. Particles were picked using EMAN2 (ref. 68); 6,863 particles were picked for Crm1, 1,128 particles for the RanBP2 complex alone and 11,957 particles for the RanBP2 complex bound to Crm1 and RanQ69L. For classification, particles were boxed out (256 × 256 pixels) without binning. For Crm1, 25 class averages (from 5,700 particles), for the RanBP2-3/4 complex alone 5 class averages (from 900 particles) and for the RanBP2-3/4 complex bound to Crm1 and RanQ69L 50 class averages (from 9,600 particles) were generated using RELION^69^ (Supplementary Fig. 2d).

## Additional information

**How to cite this article:** Ritterhoff, T. *et al*. The RanBP2-RanGAP1*SUMO1-Ubc9 SUMO E3 ligase is a disassembly machine for Crm1-dependent nuclear export complexes. *Nat. Commun.* 7:11482 doi: 10.1038/ncomms11482 (2016).

## Supplementary Material

Supplementary Information Supplementary Figures 1-4
