# Supplementary material for: The RanBP2/RanGAP1*SUMO1/Ubc9 SUMO E3 ligase is a disassembly machine for Crm1-dependent nuclear export complexes
Source: Nat Commun. 2016 May 10;7:11482. doi: 10.1038/ncomms11482 (PMC4866044; doi:10.1038/ncomms11482)
Supplement: Supplementary Information — Supplementary Figures 1-4 [file ncomms11482-s1.pdf]

Supplementary Information

Supplementary Figures

Supplementary Figure 1

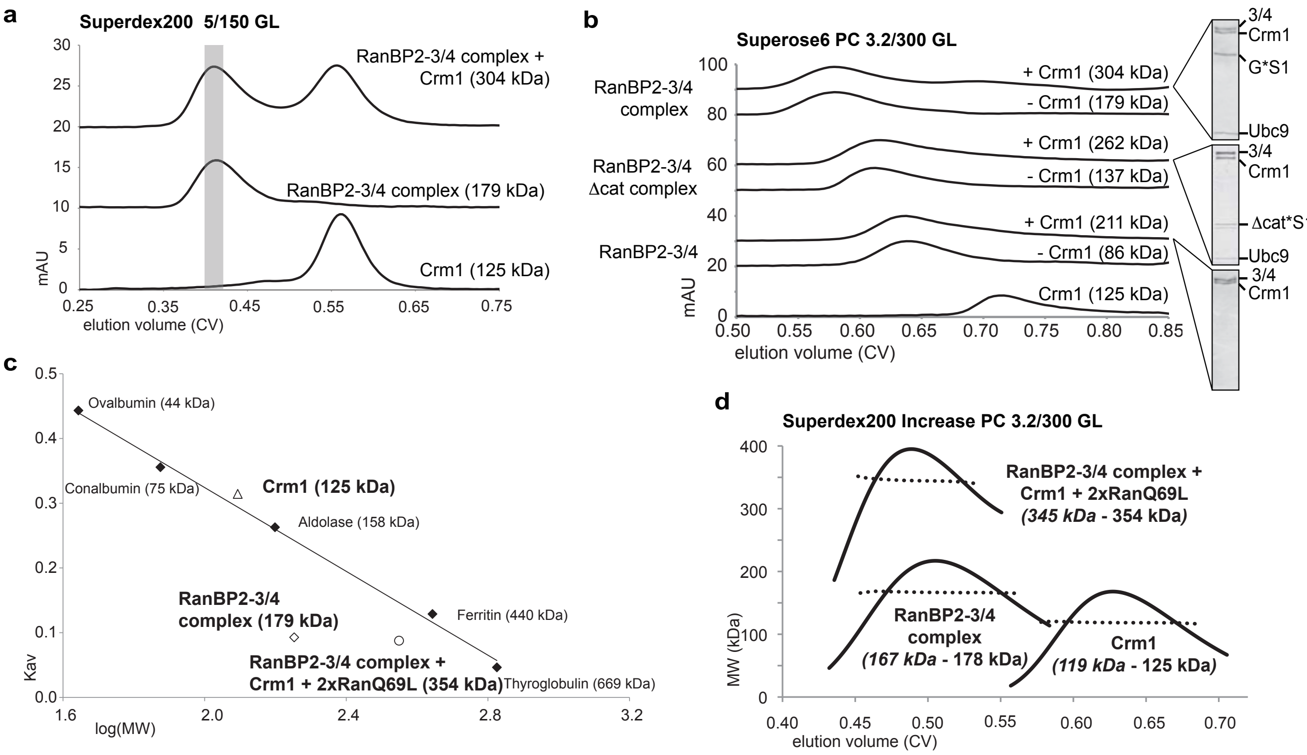

Gel filtration analysis of the RanBP2-3/4 complex bound to Crm1

(a) The RanBP2-3/4 complex bound to Crm1 can be separated from free Crm1 by gel filtration. The RanBP2-3/4 complex (1 µM) and Crm1 (2 µM) were pre-incubated separately or together on ice before they were subjected to a Superdex200 5/15 GL gel filtration column. Shown are chromatograms recorded at 280 nm. The grey box indicates the 60 μl peak fraction of the RanBP2-3/4 complex, which was typically collected for this type of binding assays. **(b)** Binding of Crm1 to RanBP2-3/4 and RanBP2-3/4 complex variants does not increase their Stokes radii despite significant increases in molecular weight. RanBP2-3/4 or RanBP2-3/4 complex variants (1 μM) and Crm1 (1.5 μM) were pre-incubated as indicated and separated over a Superose6 PC 3.2/300 GL gel filtration column. Shown are chromatograms recorded at 280 nm. Insets show peak fractions collected at 0.56, 0.61 and 0.65 CV, respectively, analyzed by SDS-PAGE and Coomassie staining. G*S1 = RanGAP1*SUMO1, Δcat*S1= RanGAP1Δcat*SUMO1, 3/4 = RanBP2-3/4. **(c)** The RanBP2-3/4 complex assumes a more compacted structure upon binding to Crm1 and RanQ69L. The RanBP2-3/4

complex (1 μM) was incubated with Crm1 (2 μM) and RanQ69L (5 μM). The binding reaction as well as Crm1 and the RanBP2-3/4 complex alone were run over a Superdex200 5/150 GL gel filtration column and elution peaks were recorded. A plot of the partition coefficient (Kav) against the log of the calculated molecular weight (log(MW)) is shown for Crm1 (triangle), the RanBP2-3/4 complex (white diamond), the RanBP2-3/4 complex bound to Crm1 and RanQ69L (circle) and a set of indicated globular standard proteins (black diamonds). **(d)** Molecular weight determination by Size Exclusion Chromatography with Multi-Angle Light Scattering analysis (SEC-MALS) of the molecular species from (a). The RanBP2-3/4 complex (4 μM) was incubated with Crm1 (8 μM) and RanQ69L (20 μM). Binding reaction as well as Crm1 and the RanBP2-3/4 complex alone were run over a Superdex200 PS 3.2/300 GL gel filtration column equilibrated in TB without protease inhibitors. Multi-angle light scattering data were collected. The graph shows the analyzed peaks from gel filtration profiles recorded at 280 nm (solid lines, not to scale) and the molecular weights from light scattering data (horizontal dotted lines to scale). Average measured values (in italic) are given next to calculated values.

Supplementary Figure 2

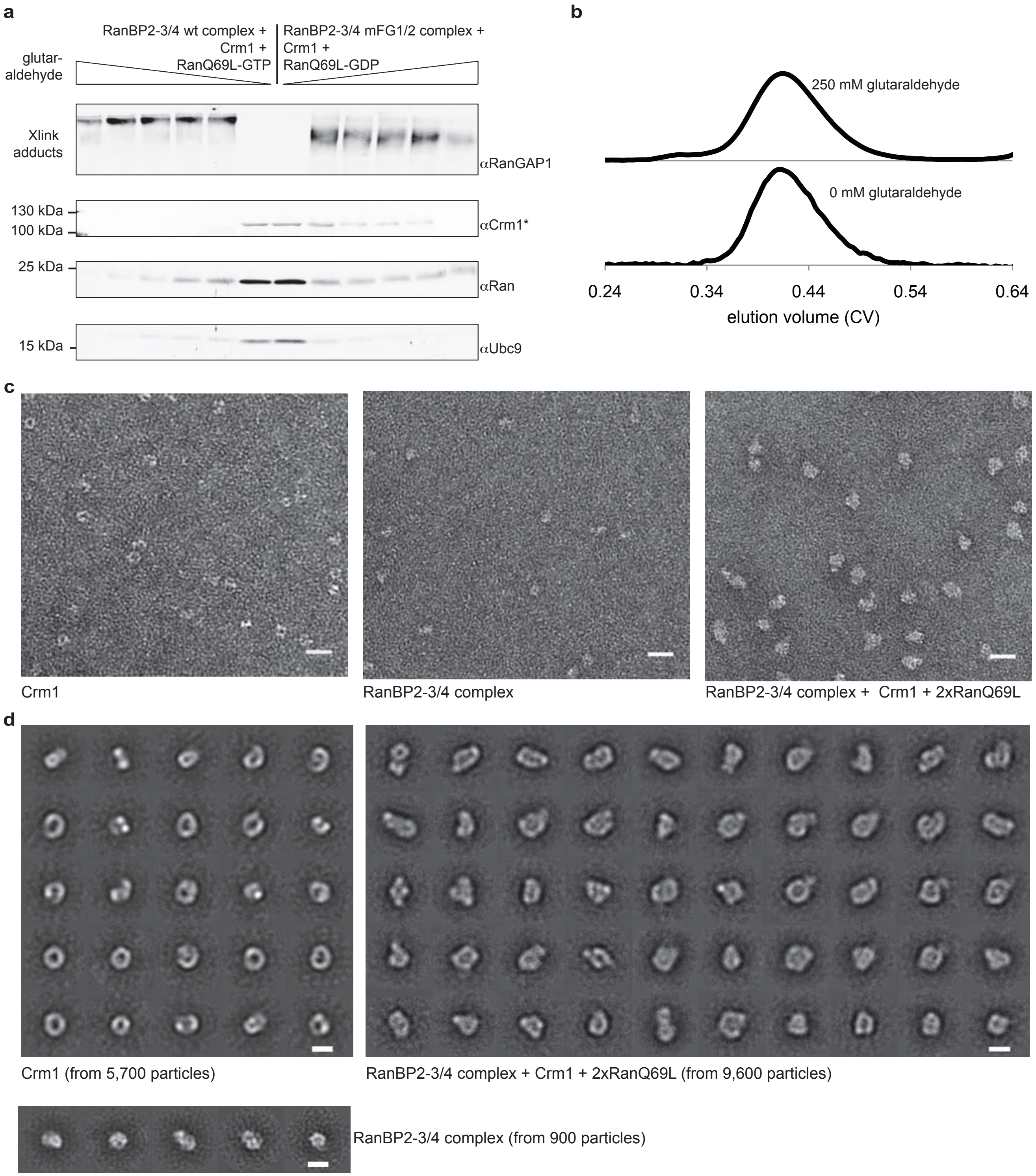

Preparation of a cross-linked RanBP2-3/4 complex bound to Crm1 and RanQ69L for EM.

Crm1 and RanQ69L-GTP can be specifically cross-linked to the RanBP2-3/4 complex. RanBP2-3/4 complex variants (400 μM), Crm1 (320 μM), RanQ69L loaded with GTP and GDP (320 μM each) were

incubated with increasing concentrations of glutaraldhyde (0, 50, 75, 125, 250 and 500 mM). Quenched cross-linking reactions (20 μl each) were analyzed for cross-linked adducts by αRanGAP1, for free Crm1 by αCrm1*, for free Ran by αRan and for free RanBP2 complex by αUbc9 immunoblotting. αCrm1* = self-made Crm1 antibody. **(b)** Glutaraldehyde cross-linking does not change the Stokes radius of the RanBP2-3/4 complex/Crm1/RanQ69L ensemble. Shown are chromatograms of a purified RanBP2-3/4 complex/Crm1/RanQ69L ensemble cross-linked and non-cross-linked (400 nM each) on a Superdex200 5/150 GL gel filtration column recorded at 280 nm. **(c)** Raw electron micrographs of uranyl acetate-stained Crm1 (top), RanBP2-3/4 complex alone (middle) and bound to Crm1 and RanQ69L (bottom); representative grid sections are shown. The RanBP2-3/4 complex alone and bound to Crm1 and RanQ69L were cross-linked with glutaraldehyde before imaging; Crm1 was imaged without cross-linking. Scale bar = 20 nm. **(d)** All 2D class averages generated for Crm1 (25 – top left), the RanBP2-3/4 complex alone (5 – bottom) and bound to Crm1 and RanQ69L (50 – top right); selected class averages are shown in Fig. 4e. Numbers of particles used to generate class averages are indicated. Scale bar = 10 nm.

Supplementary Figure 3

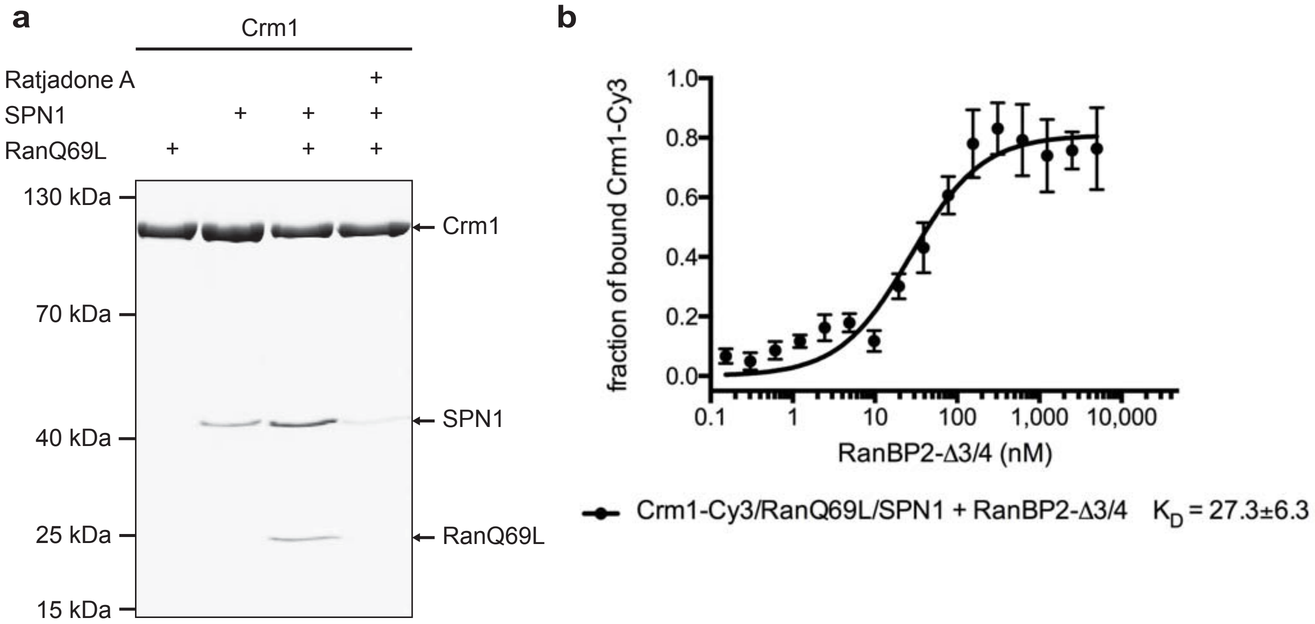

Crm1 export complex formation and binding to RanBP2 *in vitro*.

(a) Crm1 export complex formation validated by gel filtration binding assays. Ratjadone A or mock treated Crm1 (2 μM), RanQ69L (5 μM) and SPN1 (4 μM) were pre-incubated as indicated and separated over a Superdex200 5/150 GL gel filtration column. Samples collected at 0.52 CV were analyzed by SDS-PAGE and Coomassie staining. **(b)** Dissociation constant of the interaction between RanBP2-Δ3/4 and a Crm1 export complex determined by microscale thermophoresis. Recombinant Cy3-labeled Crm1 (20 nM), pre-incubated with RanQ69L (4 μM) and SPN1 (4 μM), was incubated with recombinant RanBP2-Δ3/4 fragments (0.15 nM - 5 μM) and subjected to microscale thermophoresis measurements (laser power 50-80%, LED power 20-40%). Shown is a summary of experiments with values normalized to the fraction of bound Crm1-Cy3. Error bars reflect the S.E.M. from three biological replicates.

Supplementary Figure 4

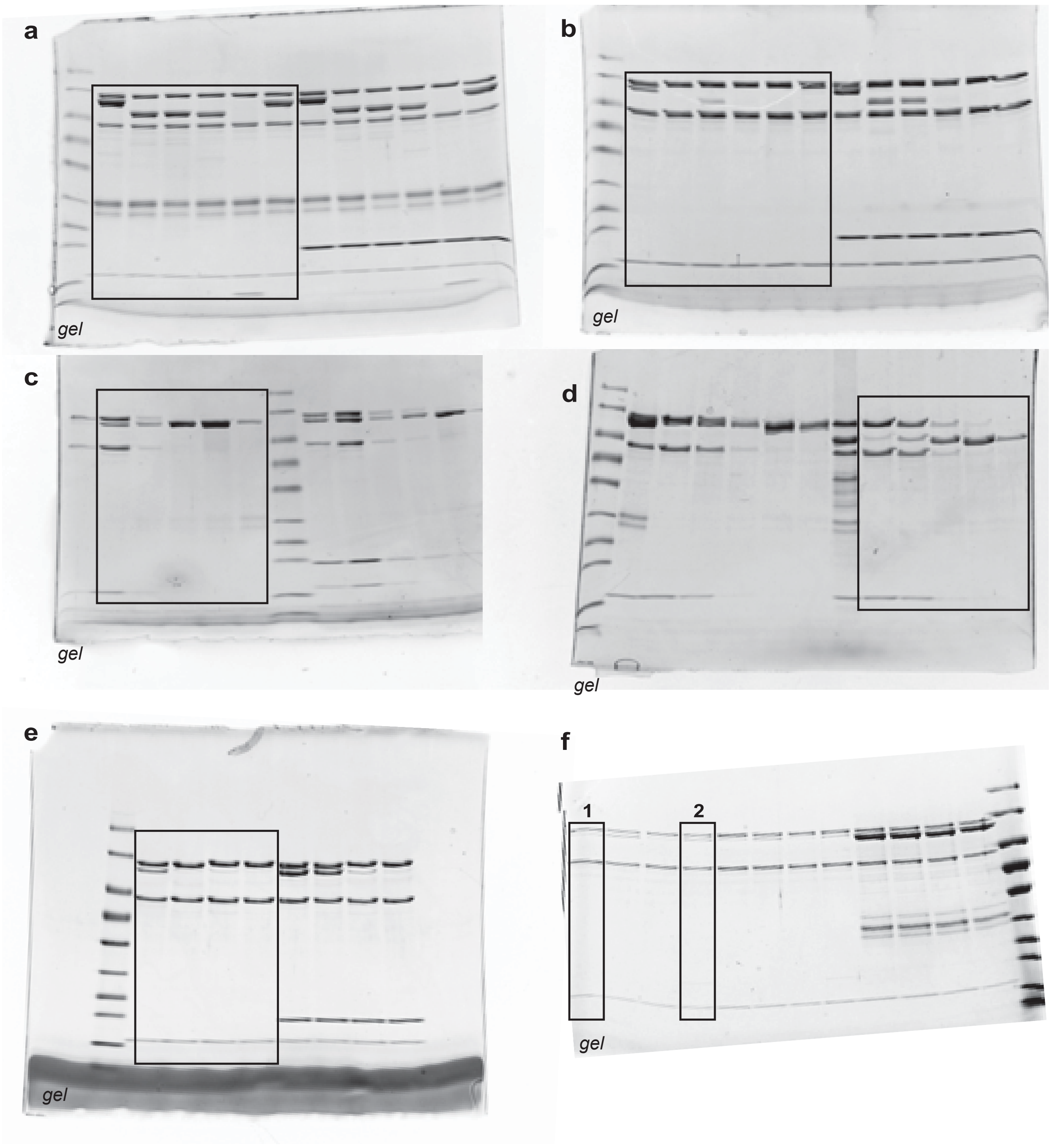

(Figure continues on next page)

(Supplementary Figure 4 continued)

g

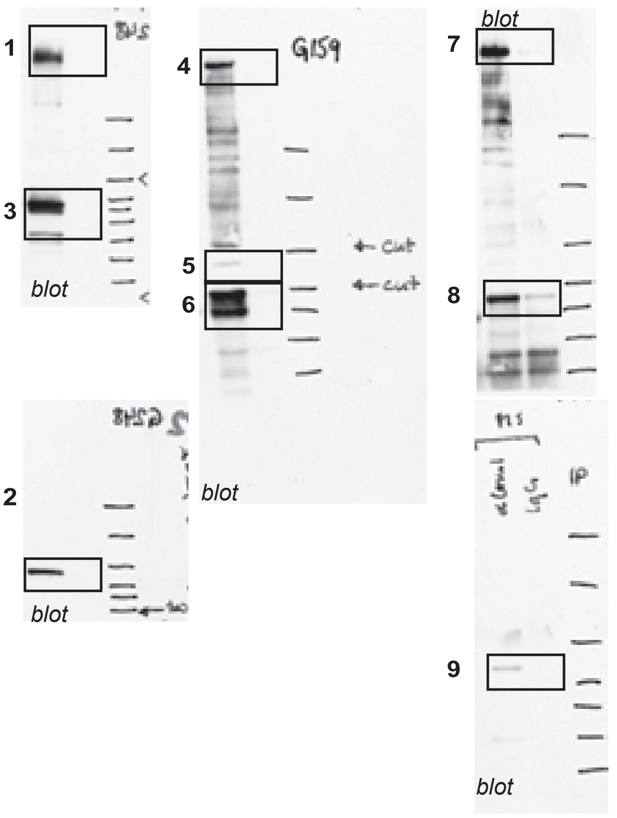

h

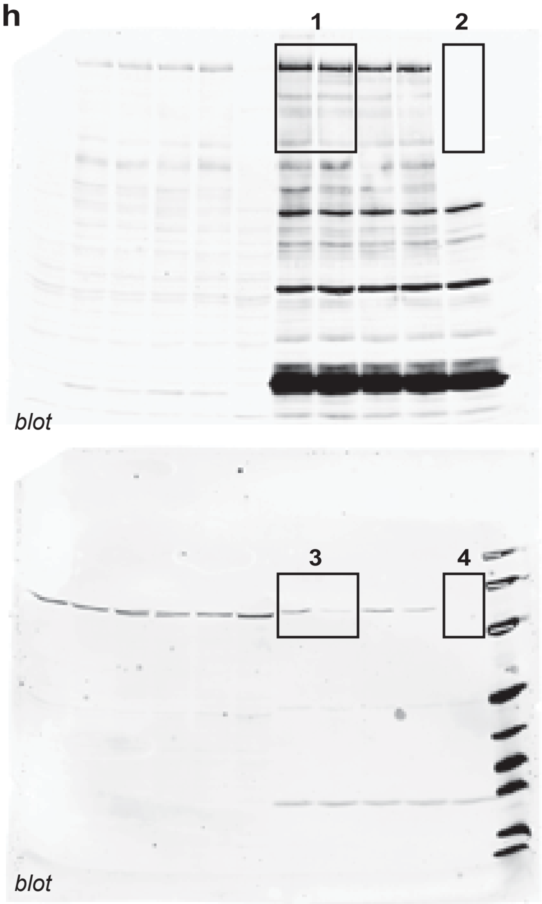

i

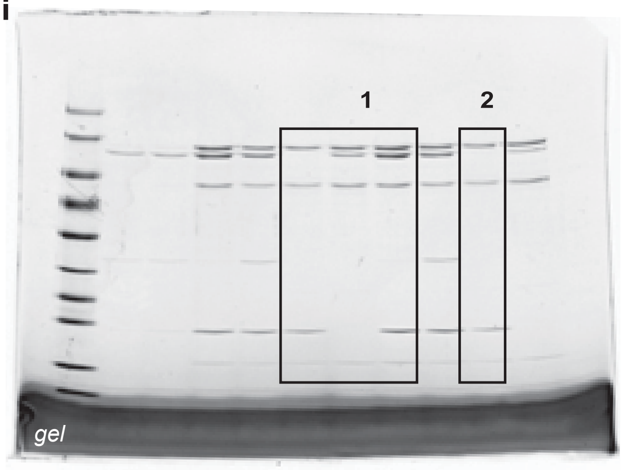

j

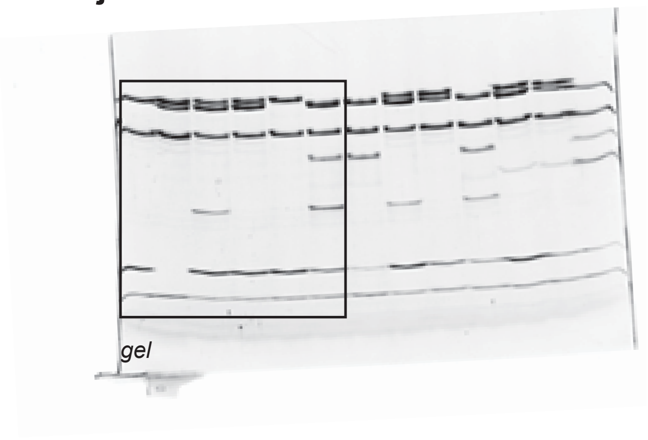

(Figure continues on next page)

(Supplementary Figure 4 continued)

k

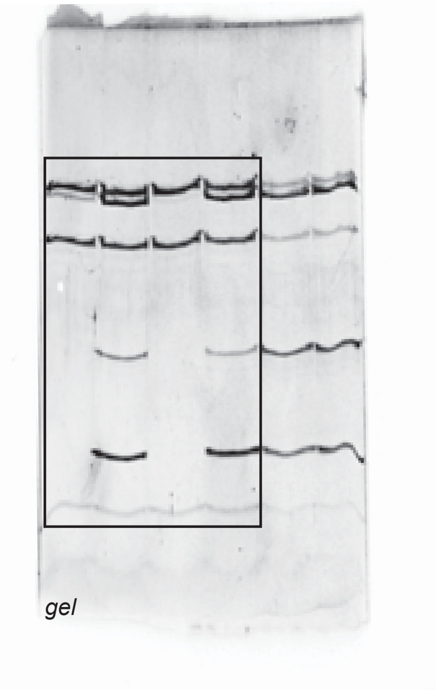

l

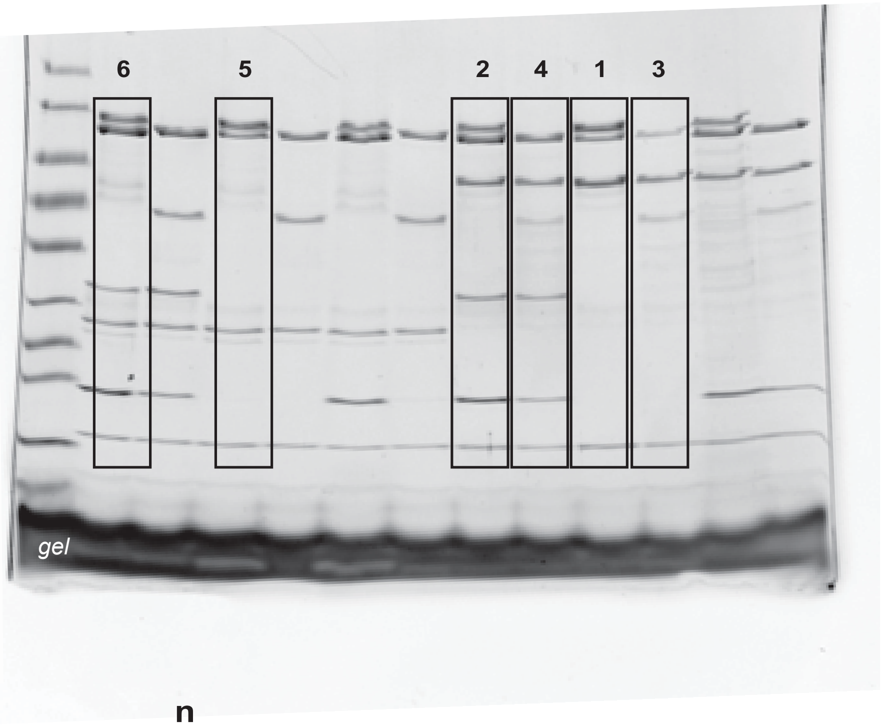

m

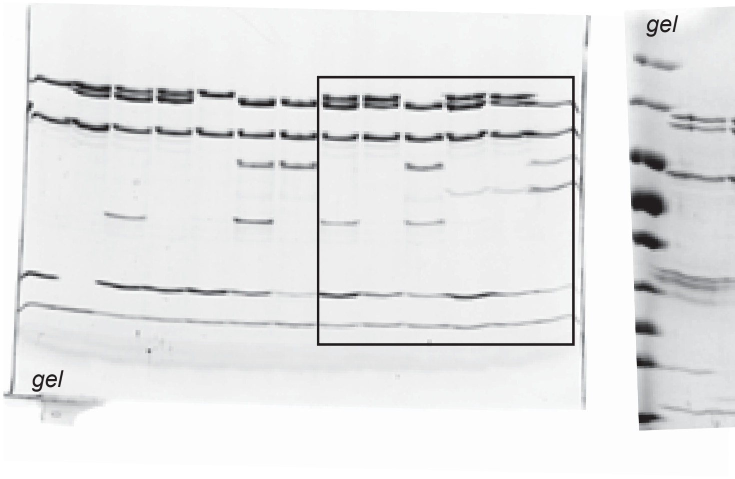

n

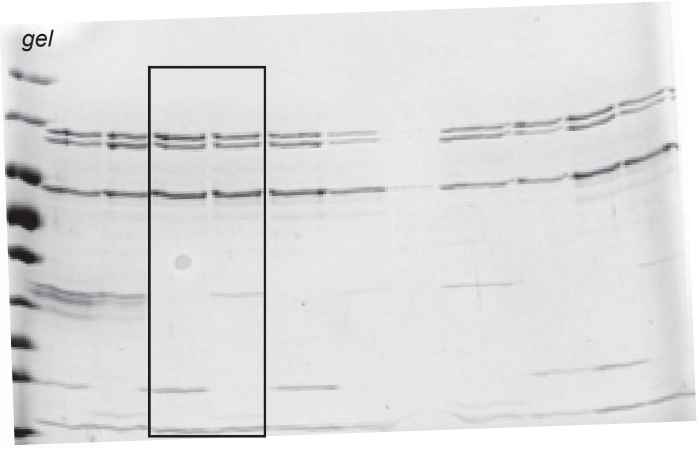

(Figure continues on next page)

(Supplementary Figure 4 continued)

o

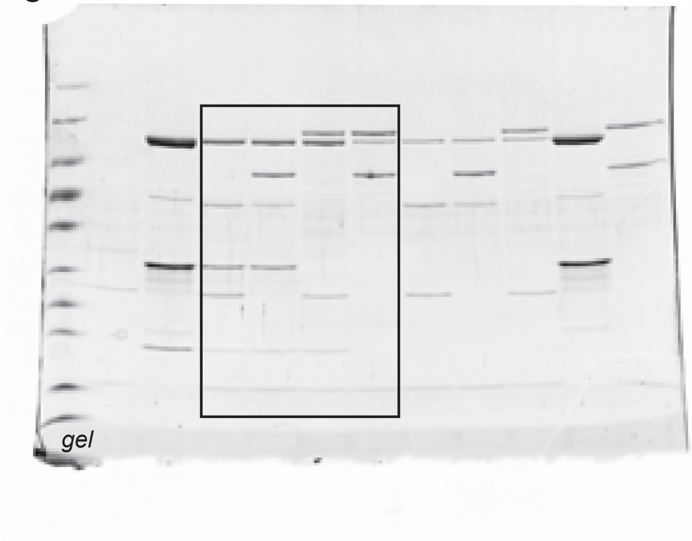

p

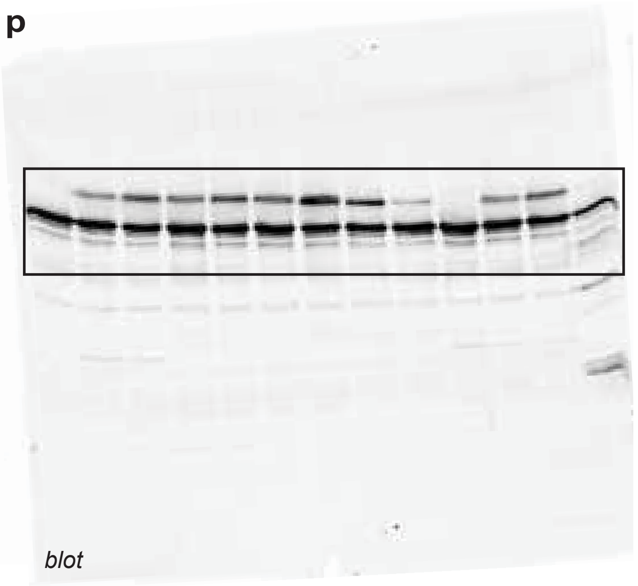

q

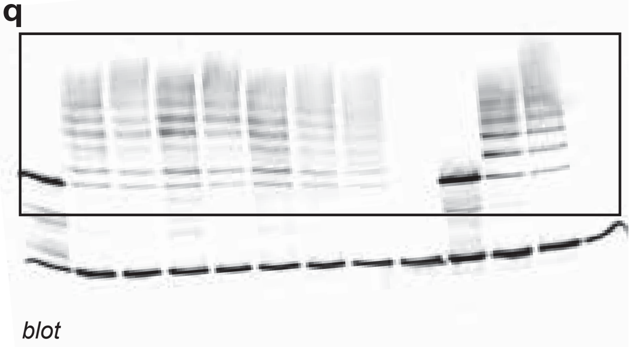

r

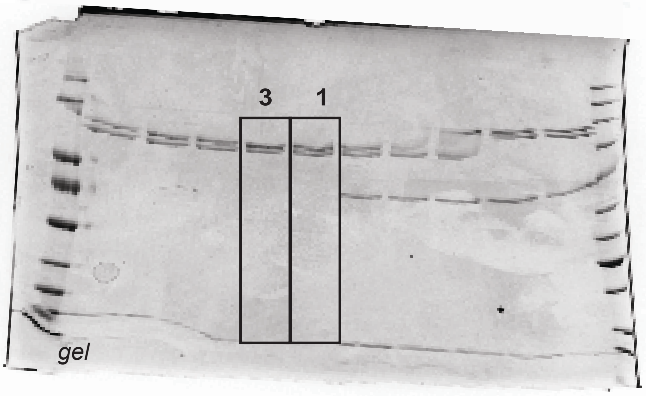

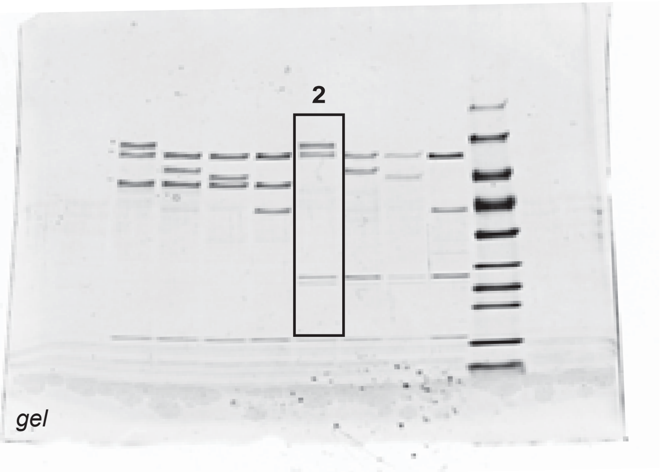

(Figure continues on next page)

(Supplementary Figure 4 continued)

s

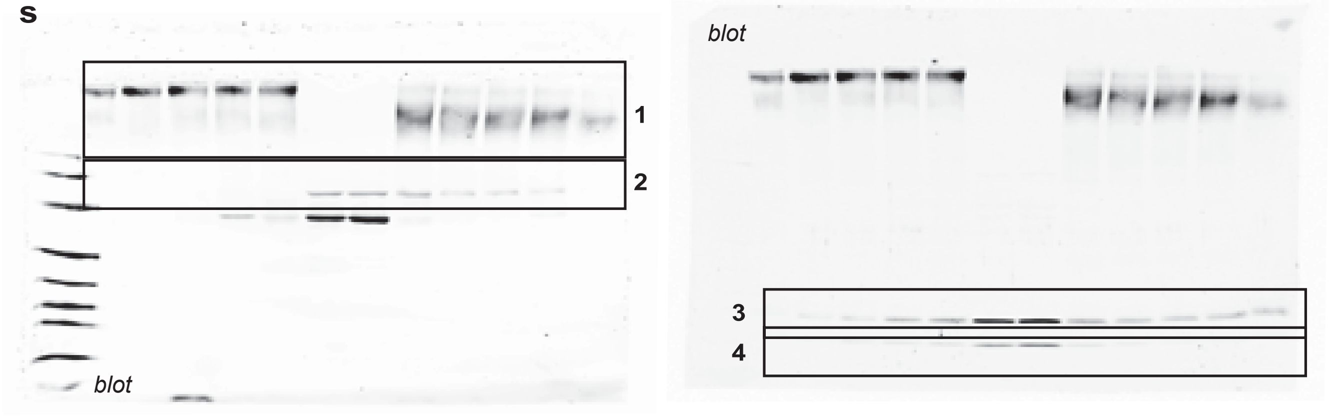

t

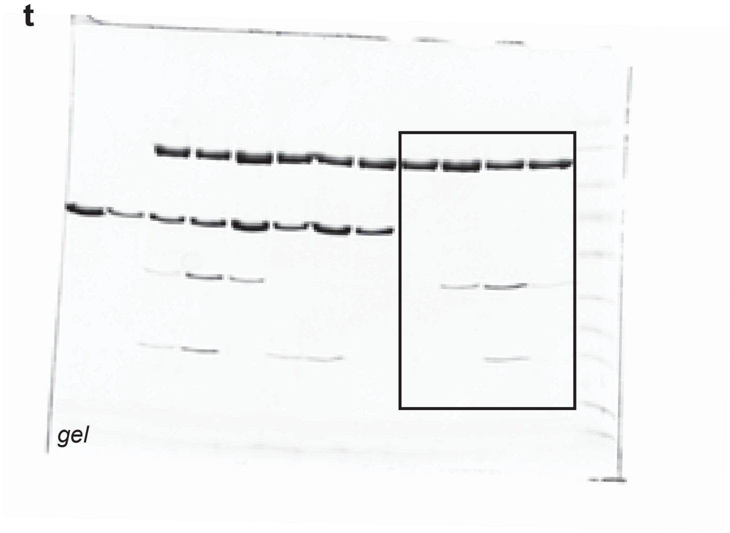

Full gels and blots from all Figures.

Uncropped Coomassie-stained SDS-PAGE gels (*gel*) and immunoblots (*blot*) from figures with boxes indicating portions of a gel/blot displayed in figures: **(a)** refers to Fig. 2a (left side), **(b)** to Fig. 2a (right side), **(c)** to Fig. 2b (left side), **(d) to** Fig. 2b (right side), **(e)** to Fig. 3b, **(f)** to Fig. 3c (1-2 referring to lanes from left to right), **(g)** to Fig. 3d (1-9 referring to lanes from left to right and then top to bottom), **(h)** to Fig. 3e (1-4 referring to lanes from left to right and then top to bottom), **(i)** to Fig. 4b (1-2 referring to lanes from left to right), **(j)** to Fig. 5a, **(k)** to Fig. 5b, **(l)** to Fig. 5c (1-6 referring to lanes from left to right), **(m)** to Fig. 6a, **(n)** to Fig. 6b, **(o)** to Fig. 6c, **(p)** to Fig. 7a, **(q)** to Fig. 7b, **(r)** to Supplementary Fig. 1b (1-3 referring to lanes from top to bottom), **(s)** to Supplementary Fig. 2 (1-4 referring to lanes from top to bottom) and **(t)** to Supplementary Fig. 3a.
